# Risk sensitivity and theory of mind in human coordination

**DOI:** 10.1371/journal.pcbi.1009167

**Published:** 2021-07-15

**Authors:** Pedro L. Ferreira, Francisco C. Santos, Sérgio Pequito

**Affiliations:** 1 INESC-ID and Instituto Superior Técnico, Universidade de Lisboa, Lisbon, Portugal; 2 Center for Systems and Control, Delft University of Technology, Delft, Netherlands; Dartmouth College, UNITED STATES

## Abstract

What humans do when exposed to uncertainty, incomplete information, and a dynamic environment influenced by other agents remains an open scientific challenge with important implications in both science and engineering applications. In these contexts, humans handle social situations by employing elaborate cognitive mechanisms such as theory of mind and risk sensitivity. Here we resort to a novel theoretical model, showing that both mechanisms leverage coordinated behaviors among self-regarding individuals. Particularly, we resort to cumulative prospect theory and level-*k* recursions to show how biases towards optimism and the capacity of planning ahead significantly increase coordinated, cooperative action. These results suggest that the reason why humans are good at coordination may stem from the fact that we are cognitively biased to do so.

## Author summary

We propose a new computational model characterizing coordination among self-regarding individuals under theory of mind and risk sensitivity. Theory of mind enables decision-making based on the attribution of beliefs, knowledge, or goals to others, whereas different risk sensitivities allows one to assess the impact of different ways of valuing uncertain returns, as captured by descriptive theories from social-economic studies. Together they provide evidence that biases towards optimism, and the capacity for planning ahead, significantly increase coordinated, cooperative action.

## Introduction

Understanding human behavior is a highly interdisciplinary endeavour—due to the complexity of the decision-making processes—with promising results in both science and engineering. Better behavioral models have been the focus of economics and psychology, often relying on mathematical frameworks used in engineering, stochastic processes, and control theory. A particularly challenging question is that of what humans do when exposed to uncertainty, incomplete information, and a dynamic environment influenced by other agents. Hereafter, we focus on studying how coordination—the process of organizing people so that they work together properly—emerges between any two agents as a result of complex human cognitive features [1]. During such processes, humans employ several mechanisms such as theory of mind and risk sensitivity [2]. *Theory of mind* is defined as one’s ability to attribute mental states (e.g. beliefs, knowledge, and goals) to others and to realize those mental states may be different from one’s own [3]. Humans are not born with this mechanism in place. Instead, we humans develop the ability to “put ourselves in other’s shoes” at around age 3, and this has been observed in fMRI experiments in children [4, 5]. In such enterprise, the level-*k* model is a model of theory of mind in which agents first assume a stereotyped behavior and progressively make use of previous behaviors to calculate more sophisticated ones in a recursive fashion [6]. Truncating the level of recursion—henceforth referred to as *k*—to a fixed level is one way to simulate the so-called *bounded rationality*.

Nonetheless, a key ingredient is missing when making decisions under uncertainty as humans might have different degrees of sensitivity to risk. On one hand, research in decision-making has long since moved away from long-held theoretical assumptions of rationality—a good example of this is the virtual bargaining model, which describes how people frame situations in terms of their worst individual outcome while attempting to evaluate what others will do [7]. On the other hand, in other areas of science, classic paradigms of decision-making under uncertainty such as expected utility theory are still in use which lack descriptive power to effectively explain and replicate human behavior [8]. *Cumulative prospect theory* (CPT) is a highly influential descriptive model of decision-making that attempts to model the different degrees of sensitivity to risk [9]. Specifically, CPT describes the risk sensitivity of people by modelling how we place value on uncertain outcomes.

Here, we propose a unified *framework to study coordination between agents equipped with both CPT and bounded rationality (up to a level-k recursion) due to the superior ability of CPT to describe human decisions and the importance of the development of a theory of mind*. Specifically, we equip agents with theory of mind and cognitive bias on risk sensitivity, and study mathematically how they coordinate in pairwise normal-form and Markov games. This approach is related with the broad literature on intention recognition, opponent modelling, and models that aim at predicting the opponents’ sequence of actions through machine learning techniques [10–12]. Here, we seek to answer the following questions:

1. Can cognitive biases concerning risk promote coordination?
2. Can increasingly sophisticated levels of theory of mind promote coordination?

We show that both of these questions are answered affirmatively. To do so, we assess the emergence of coordination with CPT, and analyze the resulting behavior with increasing *k*. Our results indicate that, while these mechanisms often create sub-optimal individual behavior, they greatly facilitate collective action in two-person scenarios. This suggests that the reason why humans are good at coordination may stem from the fact that we are cognitively biased to do so. Moreover, as we move towards a society where both humans and machines need to interact with each other and achieve coordination, we do not only need to unveil such mechanisms, but understand how to foster collective action in populations comprising humans and artificial entities [13–17].

## Results

### Risk sensitivity

In decision-making related fields, agents are assumed to have a model of which outcomes they deem valuable [18, 19]. This is done via a value function that maps outcomes to a real number, the value. CPT is used to evaluate uncertain outcomes, which are represented by a discrete random variable *R*, and it is a two step process described next.

First, the set of possible outcomes (i.e., the support of *R*) is sorted in an increasing fashion. A reference point *b* is chosen such that outcomes lower than *b* are considered losses, while outcomes higher than *b* are taken to be gains—establishing a cognitive bias known as *framing effect* [20, 21].

**Fig 1:**
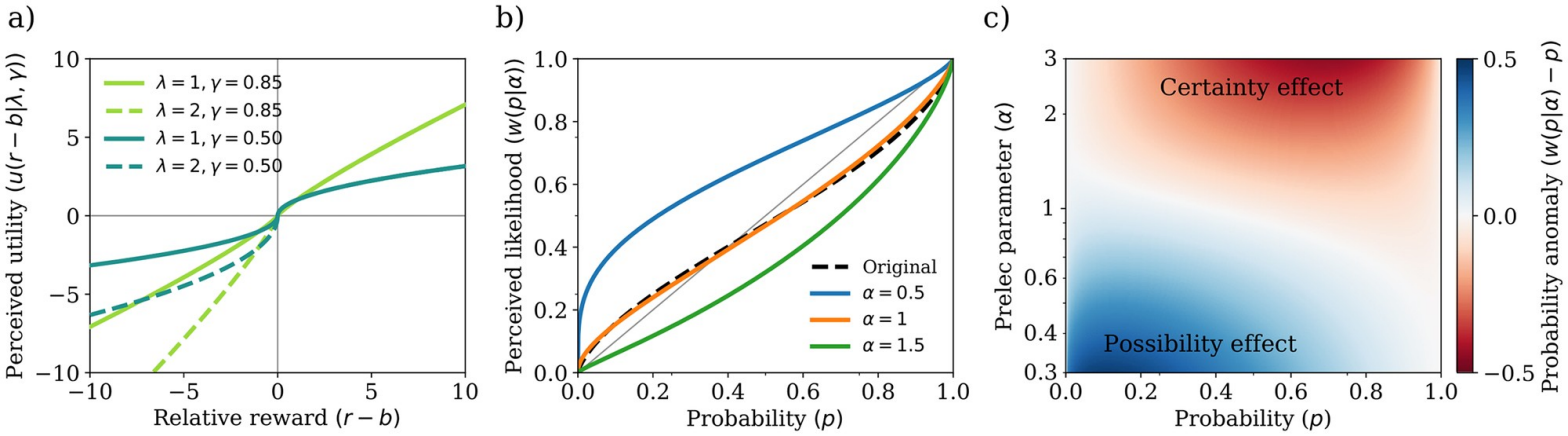
Utility and probability weighting functions used in cumulative prospect theory. (a) Utility functions for gains (*u*(*r* − *b*|*γ*) = (*r* − *b*)^*γ*^, for *r* > *b*) and losses (*u*(*r* − *b*|λ, *γ*) = −λ|*r* − *b*|^*γ*^, for *r* < *b*) used in the calculation of value under cumulative prospect theory. These are convex for gains and concave for losses, to mimic a diminishing marginal returns effect on relative rewards. Steeper utility function for losses shows loss aversion, by amplifying the perception of a loss when compared to a gain of similar magnitude. (b) Prelec’s probability weighting function, *w*(*p*|*α*, *δ*) = exp{−*α*(−log(*p*))^*δ*^}, is plotted for different values of the Prelec parameter *α* and for fixed *δ* = 0.75. The probability weighting function presented originally, $w\left(p|\gamma \right)=p^{\gamma }/{\left(p^{\gamma }+{\left(1-p\right)}^{\gamma }\right)}^{\frac{1}{\gamma }}$, is represented by the black dashed line, for *γ* = 0.85. Notice that Prelec’s function is very similar to the originally proposed probability weighting function when *α* = 1, demonstrating both overweighting of low probabilities and underweighting of high probabilities, corresponding to the *possibility* and *certainty* effects. (c) Probability anomaly, *w*(*p*|*α*) − *p*. Blue indicates positive anomaly, whereas red indicates negative anomaly. Here it is easy to see the effects of the probability weighting function; for low values of *α*, low probabilities are overweighted, causing the so-called *possibility effect*—i.e. highly unlikely events are perceived as more probable than they actually are ---, and, for high values of *α*, high probabilities are underweighted, demonstrating the certainty effect—i.e. highly likely events are perceived as less probable than they actually are. Notice that both the certainty and possibility effects come into play when *α* ≈ 1.

Second, the agent attributes *utility* to each outcome through two different functions: a utility function for gains, $u^{+}:R\to R$, and another for losses, $u^{-}:R\to R$. The parameter called reference point plays a key role as it is used to define what is a gain and what is a loss. Both utility functions show diminishing marginal returns, but the utility function for losses is steeper, describing humans’ tendency to overweight losses compared to gains of the same amount—another cognitive bias known as *loss aversion* [22, 23]. Examples of commonly used utility functions associated with CPT are displayed in Fig 1.

Furthermore, in CPT, the agent exhibits a distorted view of likelihoods captured by a probability over outcomes. The probability of outcomes is non-linearly transformed by the so-called probability weighting function, *w*: [0, 1]→[0, 1]. Naturally, this probability plays a major role in determining agents’ behavior under uncertainty in CPT. The probability weighting function of proposed originally [8] (see Methods for details) shows two cognitive biases: the *possibility effect* and the *certainty effect*. The former is an overestimation of unlikely events, while the latter is an underestimation of highly likely events. Due to numerical tractability (see Methods for further details), we use a similar function, the Prelec probability weighting function [24], *w*(*p*|*α*, *δ*) = exp{−*α*(−log(*p*))^*δ*^}, where *α* is the Prelec parameter. This choice allows us to conveniently study certainty and possibility effects by varying the Prelec parameter *α* for a fixed value of *δ*. Agents adopting high values of *α* portray the possibility effect, whereas those using one with low values of *α* show the certainty effect. Importantly, the Prelec’s function is similar to the originally proposed probability weighting function when *α* = 1, demonstrating both overweighting of low probabilities and underweighting of high probabilities, corresponding to the *possibility* and *certainty* effects—see Fig 1. These effects in probability perception therefore allow for significant deviations from optimal behavior. Translating this intuition into the mathematical framework of CPT, we first split the support of the random variable *R* representing the uncertain outcome into two sets depending on the reference point b∈R: the set of gains *O*^+^ = {*r* ∈ support(*R*):*r* ≥ *b*}, and the set of losses *O*^−^ = {*r* ∈ support(*R*):*r* < *b*}. Then we calculate the perceived likelihood of an outcome *r* as follows:

$$\begin{array}{c} \begin{array}{cc} \psi^{+}\left(r\right) & =w(P(R\geq r))-w(P(R>r))\text{,}\;\text{if}\;r\in O^{+}\text{,}\;\text{and}\\ \psi^{-}\left(r\right) & =w(P(R\leq r))-w(P(R<r))\text{,}\;\text{if}\;r\in O^{-}, \end{array} \end{array}$$ (1)

and so the value of an uncertain outcome *R*, under CPT, is instantiated by

$$\begin{array}{c} V^{\text{CPT}}\left(R\right)=\sum_{r\in O^{+}}u^{+}\left(r-b\right)\psi^{+}\left(r\right)+\sum_{r\in O^{-}}u^{-}\left(r-b\right)\psi^{-}\left(r\right). \end{array}$$ (2)

**Fig 2:**
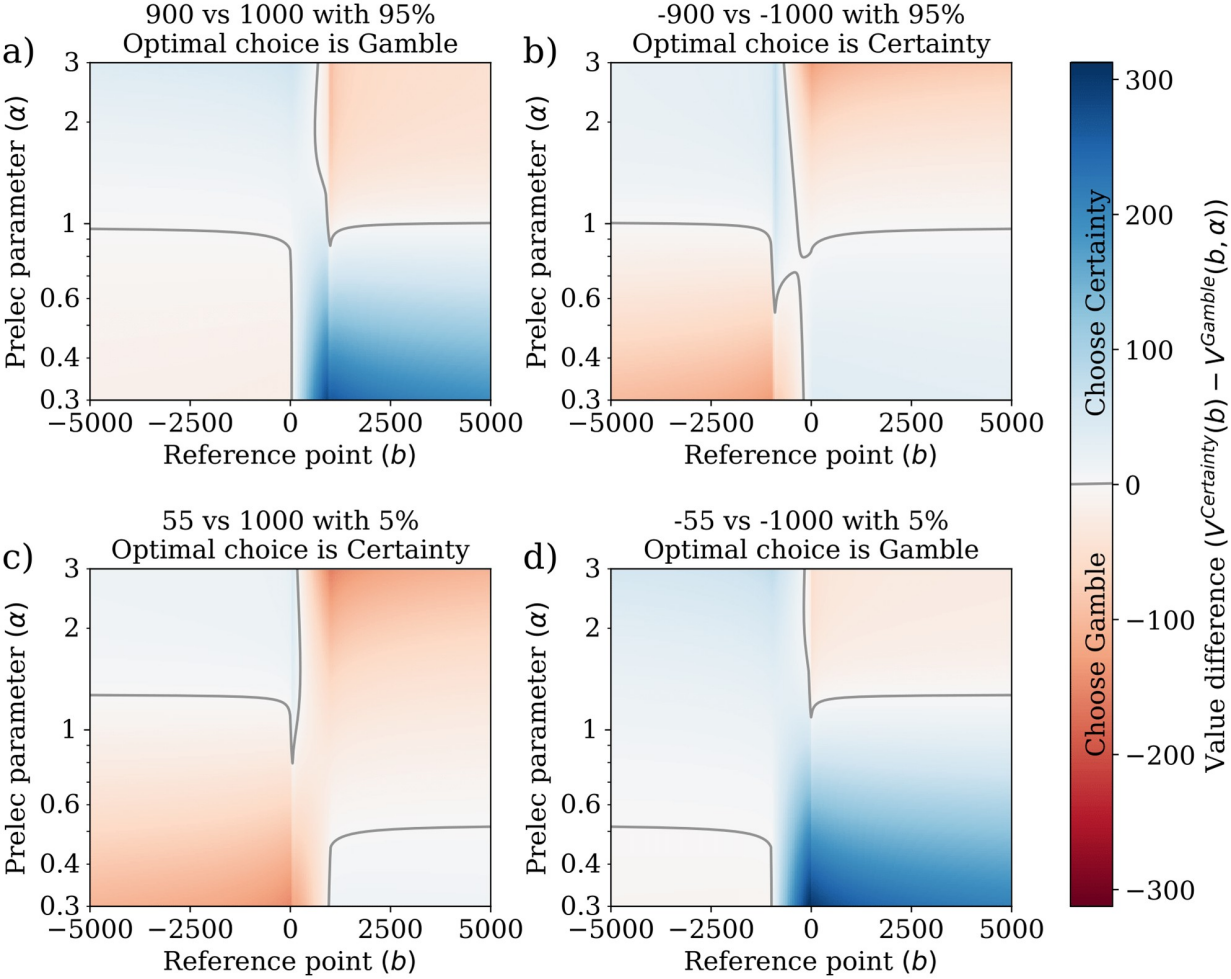
CPT-value difference between certainties and gambles, *V*^*Certainty*^(*b*) − *V*^*Gamble*^(*b*, *α*), for different reference points *b* and probability weighting functions parameterized by Prelec’s parameter *α*. The CPT-value is calculated using utility functions *u*^+^(*x*) = *x*^0.85^ for gains (*x* > 0) and *u*^−^(*x*) = −2|*x*|^0.85^ for losses (*x* < 0), and probability weighting functions are of the form *w*(*p*|*α*, *δ*) = exp{−*α*(−log(*p*))^*δ*^}, with *δ* = 0.75. Blue regions indicate larger positive differences, while red regions indicate larger negative differences. The grey solid line represents the decision boundary, where both values are equal. In (a), the agent is choosing between the certainty of gaining 900 and a gamble in which one might gain 1000 with probability 95%, where the optimal choice is ‘Gamble’ since 1000 × 95% = 950 > 900. In (b), the agent is choosing between the certainty of losing 900 and a gamble in which one might lose 1000 with probability 95%, where the optimal choice is Certainty since −1000 × 95% = −950 < −900. In (c), the agent is choosing between the certainty of gaining 55 and a gamble in which one might gain 1000 with probability 5%, where the optimal choice is ‘Certainty’ since 1000 × 5% = 50 < 55. In (d), the agent is choosing between the certainty of losing 55 and a gamble in which one might lose 1000 with probability 5%, where the optimal choice is Gamble since −1000 × 5% = −50 > −55. In all four cases, the reference point and probability perception influence behavior in significant ways. In a) and d), an optimal choice is attained when the agent either has an optimistic view of outcomes (i.e., low reference point *b*) and overestimates low probabilities (i.e., low Prelec parameter *α*), or when the agent underestimates high probabilities (i.e., high *α*) and is pessimistic (i.e., high reference point *b*). The opposite happens in b) and c). In cases where both certainty and possibility effects are present (i.e., *α* ≈ 1), behavior becomes extremely non-monotonous as a function of both parameters.

This construction of value equips agents with human-like risk sensitivity by taking into account uncertainty in decision-making. This can be seen clearly when considering the choice between a certain amount of money and a gamble. Fig 2 describes the choices of a CPT-agent, dependent on the reference point and probability distribution function, in 4 scenarios. These results suggest that (*i*) CPT can generate both optimal and sub-optimal behaviors in all scenarios, (*ii*) behavior is highly dependent on the reference point and perception of probability, especially near null reference points and when *α* ≈ 1, and (*iii*) reference point and perception of probability can switch the way one another affect decisions—for instance, if *α* is high, then increasing the reference point leads to choosing the uncertain prospect, whereas if *α* < 1, increasing the reference point has the opposite effect. A similar effect reversal happens to the Prelec parameter, when the reference point is high or low.

These different behaviors stem from the fact that CPT-agents are *risk-averse* by preferring outcomes that lead to a lower reward with a higher certainty, when faced with potentially better but uncertain outcomes. Also, the CPT-agents are *risk-seeking* by preferring uncertain outcomes that lead to a higher reward, when faced with more certain outcomes with lower reward. However, the reversal in risk attitude is highly dependent on how agents are affected by the possibility and certainty effects in their perception of probability. In other words, CPT-agents (like humans [9]) are risk-sensitive in the sense that they seek to minimize perceived losses, and that perception is highly context-sensitive.

### Theory of mind

The value of an action can be calculated as a function of the outcome that action leads to. While the risk-sensitivity notion of value makes calculations simpler, an intuitive explanation of decision-making is better done by viewing agents as choosing their behavior, and not the outcome that behavior leads to directly. In value-based models, such as CPT, agents are attempting to find the best policy *π*—a function that describes behavior—that maximizes their value, i.e.,

$$\begin{array}{c} \pi^{*}=\underset{\pi }{\text{argmax}}\;V\left(\pi \right). \end{array}$$ (3)

In a multi-agent environment, where agents make decisions simultaneously, the value of a policy may depend on the actions of the other agents. In a world with *N* agents, the value agent *i* ∈ {1, …, *N*} places on a behavior *π*_*i*_ must also be a function of the joint policy **π**_−*i*_ = (*π*_1_, …, *π*_*i*−1_, *π*_*i*+1_, …, *π*_*N*_) of all the other agents in the world, which can be written as *V*_*i*_(*π*_*i*_, **π**_−*i*_), where *V*_*i*_ is the value function of agent *i*. In summary, each agent will attempt to choose the behavior that maximizes his value function, given the behavior of the other agents. Mathematically, given **π**_−*i*_, agents perform the following optimization:

$$\begin{array}{c} \pi_{i}^{*}=\underset{\pi_{i}}{\text{argmax}}\;V_{i}\left(\pi_{i},\pi_{-i}\right). \end{array}$$ (4)

For instance, in a two-agent scenario, the simultaneity assumption demands that both agents must somehow guess which behavior the other will decide upon in order to perform the maximization of value. The level-*k* bounded rationality model [6] provides a description of the way humans do this, by assuming that each agent holds a stereotypical belief about the behavior of the other agent, which allows them to maximize value under that assumption. By also assuming each agent also maintains a stereotyped behavior of itself—as seen by others—this allows them to create a tower of increasingly sophisticated policies, up to some level-*k* as follows:

$$\begin{array}{c} \begin{array}{cc} \pi_{2}^{\left(0\right)}\; & \pi_{1}^{\left(0\right)}\\ ↓\; & ↓\\ \underset{\pi_{1}}{\text{argmax}}\;V_{1}\left(\pi_{1},\pi_{2}^{\left(0\right)}\right)=\pi_{1}^{\left(1\right)}\; & \pi_{2}^{\left(1\right)}=\underset{\pi_{2}}{\text{argmax}}\;V_{2}\left(\pi_{1}^{\left(0\right)},\pi_{2}\right),\\ \vdots \; & \;\vdots \\ \underset{\pi_{1}}{\text{argmax}}\;V_{1}\left(\pi_{1},\pi_{2}^{\left(k-1\right)}\right)=\pi_{1}^{\left(k\right)}\; & \pi_{2}^{\left(k\right)}=\underset{\pi_{2}}{\text{argmax}}\;V_{2}\left(\pi_{1}^{\left(k-1\right)},\pi_{2}\right). \end{array} \end{array}$$ (5)

The diagram above depicts the recursive reasoning in the level-*k* bounded rationality model. Starting from the top, both agents assume level-0 policies $\pi_{1}^{\left(0\right)}$ and $\pi_{2}^{\left(0\right)}$—called stereotyped policies. With these, both agents can calculate their level-1 policies and, by assuming the stereotyped policies are common knowledge—or, perhaps more reasonably, both agents are similar in such a way that they regard others as if they were themselves—they can calculate each other’s level-1 policies. The process can repeat itself up to some level-*k*. Note that it may be the case that the stereotyped policies are wrong, or that $\pi_{1}^{\left(0\right)}$ is different for agent 1 and for agent 2 (i.e., what agent 1 believes agent 2 believes agent 1 will do is not the same as what agent 2 believes agent 1 is doing). Although these are interesting questions to pose, we will not discuss these cases here, and focus instead on the effects this hierarchy of behaviors has on coordination among agents with theory of mind.

In what follows, we analyze the stag-hunt game that describes the interaction between individuals when they are given a choice between a safe but low payoff outcome and a risky but high payoff outcome [25]. Specifically, we consider both the normal-form and the Markov game versions played by agents that measure value using CPT and that are equipped with level-*k* bounded rationality.

### Hunting stags with risk-sensitive hunters and the emergence of coordination

The stag-hunt game is a well-known two-agent coordination game in which two agents (hunters) must choose between a safe option with low payoff outcome (hunting a hare), and a risky alternative with high payoff outcome (hunting a stag). The stag-hunt game has been one of the most studied coordination games due to the strong analogy between the theoretical setting and real-world conflicts [25]. The stag-hunt game is a symmetric normal-form game with two agents *N* = {1, 2}, each with two actions *A*_1_ = *A*_2_ = {*S*, *H*}, where *S* stands for *Stag* and *H* stands for *Hare*—see Section 2 of S1 Text for a brief review of normal-form games. Throughout this paper, we shall consider the utility functions presented in Table 1.

**Table 1:** Payoff matrix underlying the utility functions of the 2-player stag hunt game with two actions—Hare (H) and stag (S). Specifically, agent 1’s choices are cast in rows and agent 2’s choices are cast in columns. The utility given to agent 1 and agent 2 are the first and second numbers, respectively, of a given cell.

		Agent 2	
		*S*	*H*
Agent 1	*S*	5, 5	−1, 1
	*H*	1, −1	1, 1

The Nash equilibrium of a stag-hunt game between CPT-agents is a useful tool to analyze the effects of risk sensitivity in social settings. In the stag-hunt, there exist two pure Nash equilibria: both hunters choose to hunt stags, or both hunters choose to hunt hares. However, no notion of value has to be conjured to obtain this result and, to identify the effects of risk sensitivity on social decision-making, the mixed Nash equilibrium offers an alternative route.

If both hunters use CPT to evaluate their actions, then a mixed equilibrium can be found by identifying the probability of choosing to hunt the stag, p*, that makes the value of either action be equal, i.e.,

$$\begin{array}{c} V^{\text{CPT}}\left(S|p^{*},\alpha ,b,\lambda ,\gamma \right)=V^{\text{CPT}}\left(H|p^{*},\alpha ,b,\lambda ,\gamma \right), \end{array}$$ (6)

where *α* is the Prelec parameter [24] that describes the perception of probability, *b* is the reference point that describes how outcomes are framed, λ describes the hunter’s aversion to loss, and *γ* describes concavity of the utility function, leading to diminishing marginal utility effects. Notice that we are assuming both agents have an equal perception of probability, utility, and reference point. This simplifies the analysis to a manageable number of degrees of freedom while still allowing for complex behavior to be studied.

**Fig 3:**
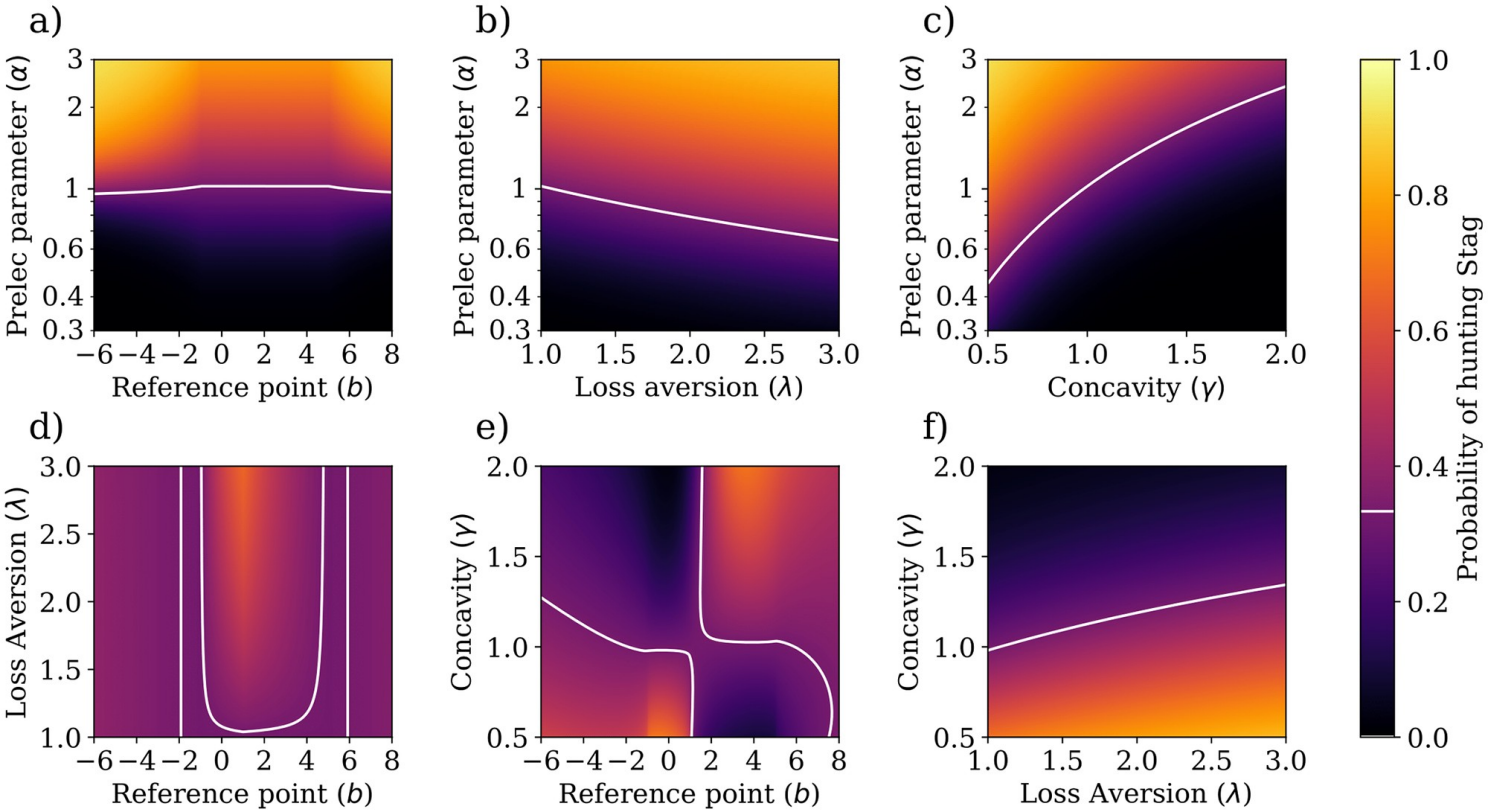
Probability of hunting stags in a normal-form stag hunt game. Cumulative prospect theory can explain a wide range of coordinating behaviors in a simple game such as the stag hunt, depending on how probabilities are perceived and how outcomes are framed. The panels show the probability of choosing Stag in the stag hunt normal-form game, for pairs of parameters of prospect theory—i.e., reference point *b*, Prelec parameter *α*, loss aversion λ, and utility concavity *γ*. For each pair of parameters, the remaining ones were left at default values: *b* = 0, *α* = 1, λ = 1, and *γ* = 1. Lighter colors indicate higher probability of hunting stag. White line represents the mixed Nash equilibrium of 1/3.

Fig 3 shows the probability of choosing to hunt the stag for different Prelec parameters (i.e., *α*, reference points *b*, loss aversions λ, and utility concavity *γ*). It is worth noting that while we call *γ* the utility concavity, we have also analyzed the behavior of the hunters for *γ* > 1, meaning that the utility function is actually convex in that interval. However, this inclusion of convex utility functions should not speak to the validity of increasing marginal utility hypotheses.

In Fig 3a, we observe a high dependence on perception of probability, in line with the previous analysis of a simple gamble. However, here we see how this risk-related cognitive bias affects coordination and, hence, collective welfare. When hunters value their actions according to CPT, coordination is successful either when low probabilities are overestimated (i.e., low *α*), or when high probabilities are underestimated (i.e., high *α*). These correspond to regions where the probability of hunting Stag is higher than the standard mixed Nash equilibrium of *p*_Nash_ = 1/3, which corresponds to an expected collective reward of *R*_Nash_ = 2. The apparent irrelevance of the reference point in this analysis does not mean the reference point does not affect coordination, as illustrated by Fig 3d and 3e.

Fig 3b and 3c show how coordination changes with loss aversion and the diminishing of marginal utility, when paired with changes in the perception of probability. In both cases, a high Prelec parameter *α* leads to increased coordination. However, high loss aversion promotes this effect by perceiving the hare solution as worse than it actually is, whereas utility function concavity does the opposite by decreasing the perceived utility of the stag more than it does with the hare. In fact, Fig 3f shows that utility concavity and loss aversion have clear opposite effects on coordination.

Similarly, in Fig 3d, we observe that coordination is highly dependent on the reference point, and that increased loss aversion can improve coordination when the reference point is close to zero. It is important to note that the differences outside this range seems not very significant since the probability of hunting stags is very close to the Nash equilibrium—noticed by the close-to-uniform colors of the plot.

Fig 3e shows the effects of the reference point and utility concavity on coordination. For concave utility functions *γ* < 1, there is a region of positive reference points which makes it difficult for hunters to coordinate. On the other hand, when the utility function is convex *γ* > 1, the region is (mostly) is mostly in the negative part of the reference point, and is larger than its concave counterpart.

Henceforth, it readily follows that CPT can generate a wide range of two-agent coordinating behaviors that can be studied under the light of game theory with a sufficiently low number of degrees of freedom while allowing for their visualization, and capturing representative behaviors from real-life scenarios.

Notwithstanding, humans are rarely faced with scenarios where only a single decision is available. Dealing with decisions over time, dynamic environments, other people, and limited information, are what humans excel at throughout their lifetimes. To study risk sensitivity and how humans model other humans (i.e., theory of mind), we set up a Markov game [26] based on the stag-hunt game—similar to the stag-hunt in [27]. Here, two agents can be in one of 16 states ($S_{1}=S_{2}=\left\{0,...,15\right\}$, such that the joint state space is $S=S_{1}\times S_{2}$), starting in one of them at random. These 16 states represent areas within a hunting region, on the bottom of a long canyon.

**Fig 4:**
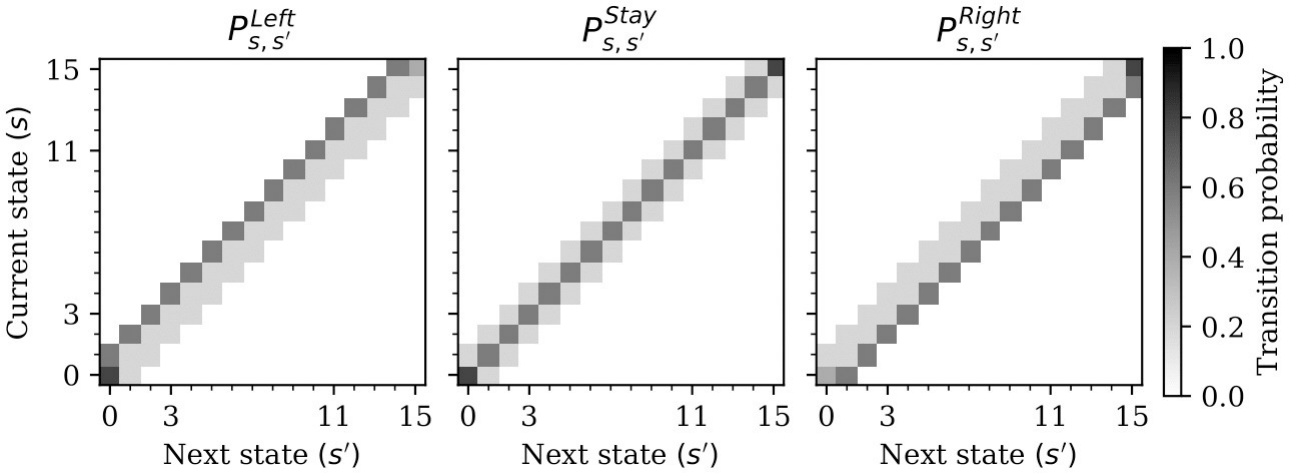
Markov stag-hunt transition probabilities of an individual agent. Darker colors indicate higher probability. Shown are 5 different colors, which, from lightest to darkest, have probabilities of 0% (white), 20%, 40% (corners), 60%, and 80% (corners). Black does not appear because there are no degenerate transitions which would make agents get stuck. Each agent chooses one of three actions (Left, Stay, or Right) and, depending on their current state, move to another state according to the respective transition probabilities. The state of an agent does not change the transition probabilities of the other agent, e.g. an agent cannot block the other agent.

**Fig 5:**
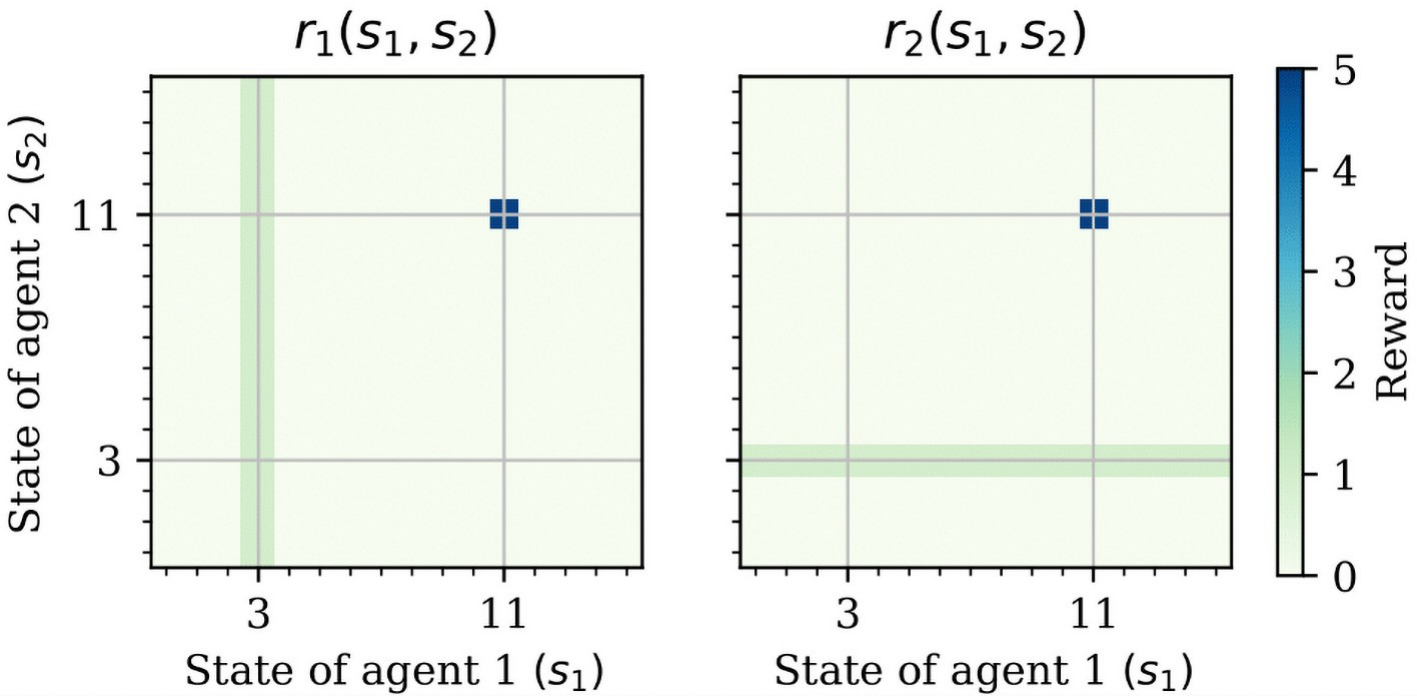
Markov stag-hunt reward functions. Darker colors indicate larger rewards. Agents receive a reward at each time step depending on their state and the state of the other agent. The hare state (state 3) can be obtained regardless of where the other agent is. This also allows us to model situations in which an agent can only obtain a big reward if the other agent is willing to coordinate with him. In our case, the stag state (i.e., state 11) has one such big, but difficult to obtain reward.

Each agent *i* = 1, 2 can move around in this canyon, by choosing an action *a*_*i*_ from their action set $A_{i}$ (with $A_{1}=A_{2}=\left\{Left,Stay,Right\right\}$). These actions have some probability to fail, in which case they may still go to in the desired direction, but can also stay at the current location, or go in the opposite direction—see Fig 4. In this canyon, only two of the 16 states have prey. Specifically, in state 3 there are hares and in state 11 there are stags. A single hunter can hunt hares alone, but coordination between the hunters is required in order to hunt stags—see Fig 5 for a summary.

Solving a Markov game results in finding the joint policy resultant from each agent minimizing their value. The notion of value here is defined as the discounted sum of rewards which can be rewritten recursively as a Bellman equation [28]. This sum occurs over the temporal trajectory of the agent, which is here assumed to be infinite. To make sure this sum stays finite, a parameter called *discount factor*, denoted by *β* ∈ (0, 1), describes how an agent values a short-term reward over a long-term one. Specifically, increasing *β* increases the perceived “goodness” of long-term rewards over short-term outcomes, while decreasing *β* makes the agent more hedonistic by concerning itself less with long-term outcomes. A Bellman equation [28] for the CPT-value has been studied before [29, 30]. Here, we generalize this notion to the multi-agent setting of a Markov game—see Section 3 of S1 Text for a detailed summary of the Markov game framework, the calculation of CPT-value, and the level-*k* bounded rationality model. In the following experiments, both agents assume uniform stereotyped policies. In other words, it is common knowledge to both agents that, at the *sophistication* level *k* = 0, each action is equally likely to be chosen by either agent in any of the 16 × 16 joint states.

#### Analysis of value and policy

**Fig 6:**
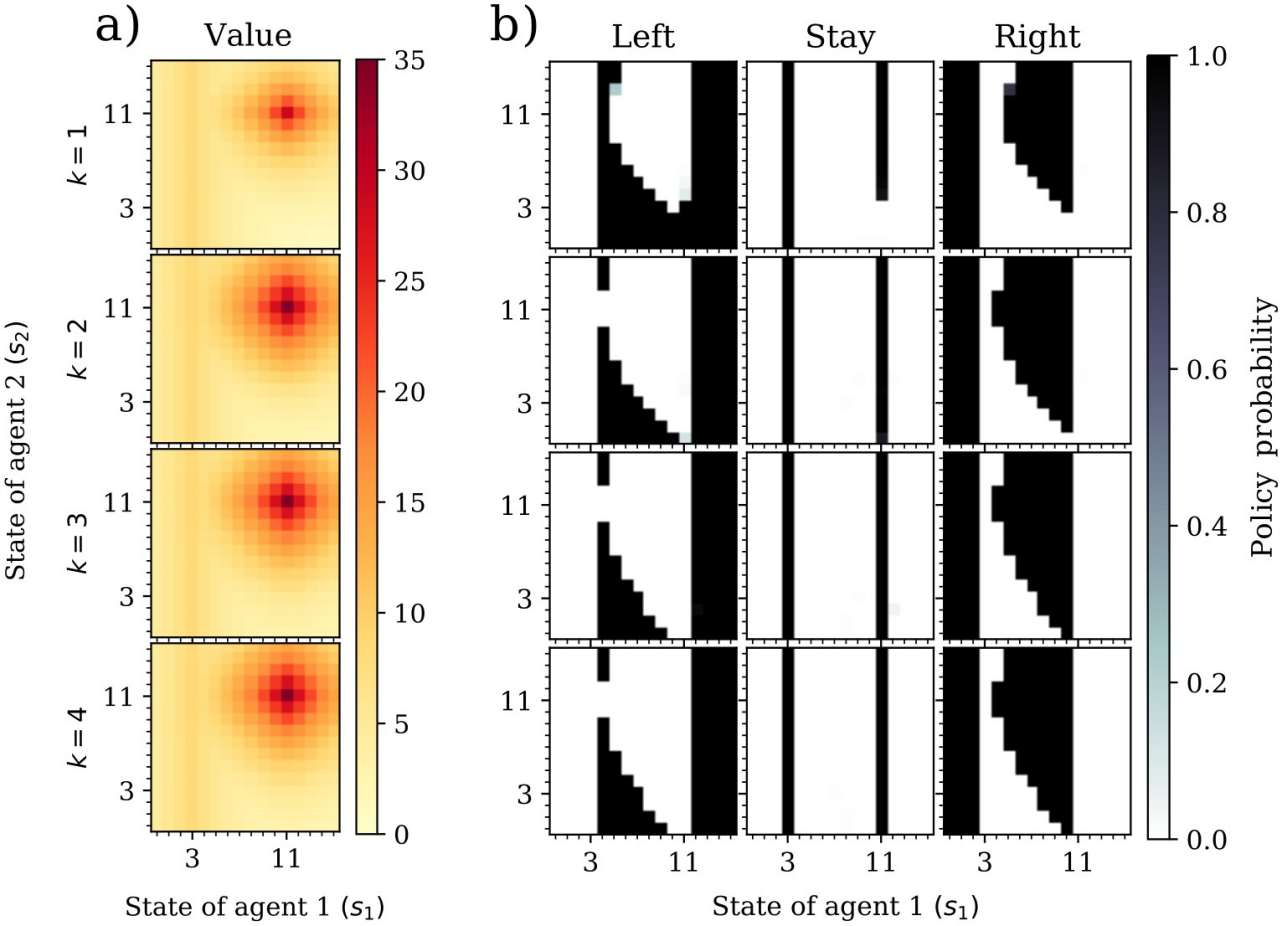
Effect of sophistication in value and policies in the Markov stag hunt game. (a) CPT values of the agent states, for sophistication levels *k* = 1, 2, 3 and 4. Joint states with redder colors have higher value. (b) Policies for sophistication levels *k* = 1, 2, 3 and 4. Joint states with darker color indicate higher probability. The value of the stag state grows with the sophistication level. We assumed reference points *b*_1_ = *b*_2_ = 0, discount factors *β*_1_ = *β*_2_ = 0.9, utility function *u*(*x*) = *x* and probability weighting $w\left(x\right)=e^{-0.5{\left(-\log\left(x\right)\right)}^{0.9}}$.

Fig 6 shows the value and policy of a hunter in a Markov game with two hunters using CPT. In this scenario, with reference point at zero, CPT-agents place increasingly more value on the stag state as the common sophistication level increases. The corresponding policies also prescribe a behavior which tends to increasingly move toward the stag state as sophistication increases. For example, from *k* = 1 to *k* = 2, hunters will choose to stay on the stag state even if the other agent is very far away from the stag. In addition, as the sophistication level increases, the number of neighboring joint states that attract hunters toward the stag state increases. Hence, theory of mind has a positive effect on coordination and steers hunters to the best outcome, something which could not be possible in the classical setup without an additional mechanism.

#### Analysis of stationary distribution

**Fig 7:**
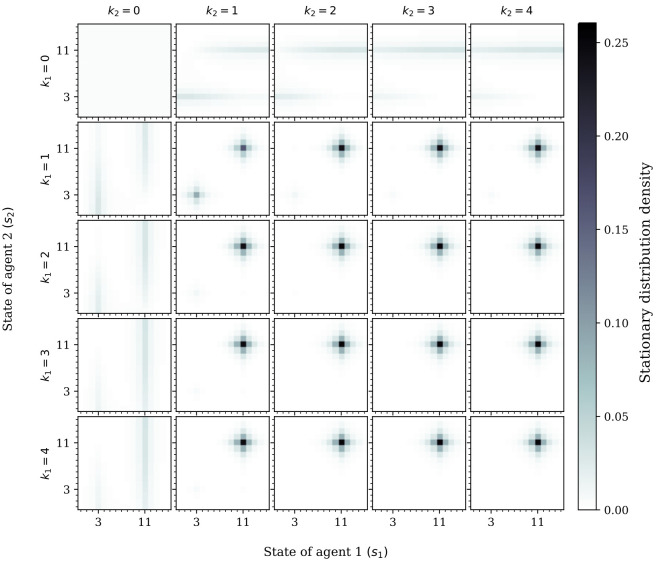
Role of sophistication level of CPT-agents in the stationary distributions of the Markov stag hunt game. Darker colors indicate higher probability. We assumed equal agent parameters (i.e., discount factors *β*_1_ = *β*_2_ = 0.9, reference point *b*_1_ = *b*_2_ = 0, utility functions *u*_1_(*x*) = *u*_2_(*x*) = *x*, and weighting function $w_{1}\left(x\right)=w_{2}\left(x\right)=e^{-0.5{\left(-\log\left(x\right)\right)}^{0.9}}$).

We can further study human behavior, at some sophistication level *k*, by capitalizing on the stationary distribution of the Markov chain that results from conditioning the Markov game’s transition probability function on a joint policy. The stationary distribution *ρ*(*k*) indicates the likelihood (in the long-run) of finding the agents in a certain state—when agents use $\pi_{1}^{\left(k\right)}$ and $\pi_{2}^{\left(k\right)}$ (i.e., the same policy at *k*-level of bounded rationality)—see Methods for a more detailed explanation of the stationary distribution. Fig 7 shows the stationary distribution for two CPT-agents playing the Markov stag-hunt game. Our results suggest that, with increasing sophistication level *k*, *CPT-agents are able to coordinate (i.e., choose the optimal stag state)* with increasing sophistication level, when assuming uniform stereotype policies. Remarkably, it seems that there is no need for a high sophistication level for coordination to emerge; in fact, coordination is at its maximum when only one of the two agents has sophistication level equal to *k*_1_ = 2 (or higher), while the other has a simpler sophistication level of *k*_2_ = 1. Recall that *k* = 0 means the agent has a uniform policy, and therefore, it cannot be expected to coordinate effectively at that sophistication level. Furthermore, note that when *k*_2_ = 1 and *k*_1_ > 2, agent 1 is overestimating the sophistication level of agent 2. Increasing *k*_1_ further increases the “error” in its assumption about agent 2. For this reason, it is surprising that coordination is not only attained, but even more so. One possible reason for this is that the assumption “error” for large *k*_1_ induces a policy of agent 2 which prefers stags with higher and higher likelihood than the previous sophistication level (i.e., *k*_1_ − 1), leading to an ever-increasing preference to hunt stags.

#### Analysis of reference point

**Fig 8:**
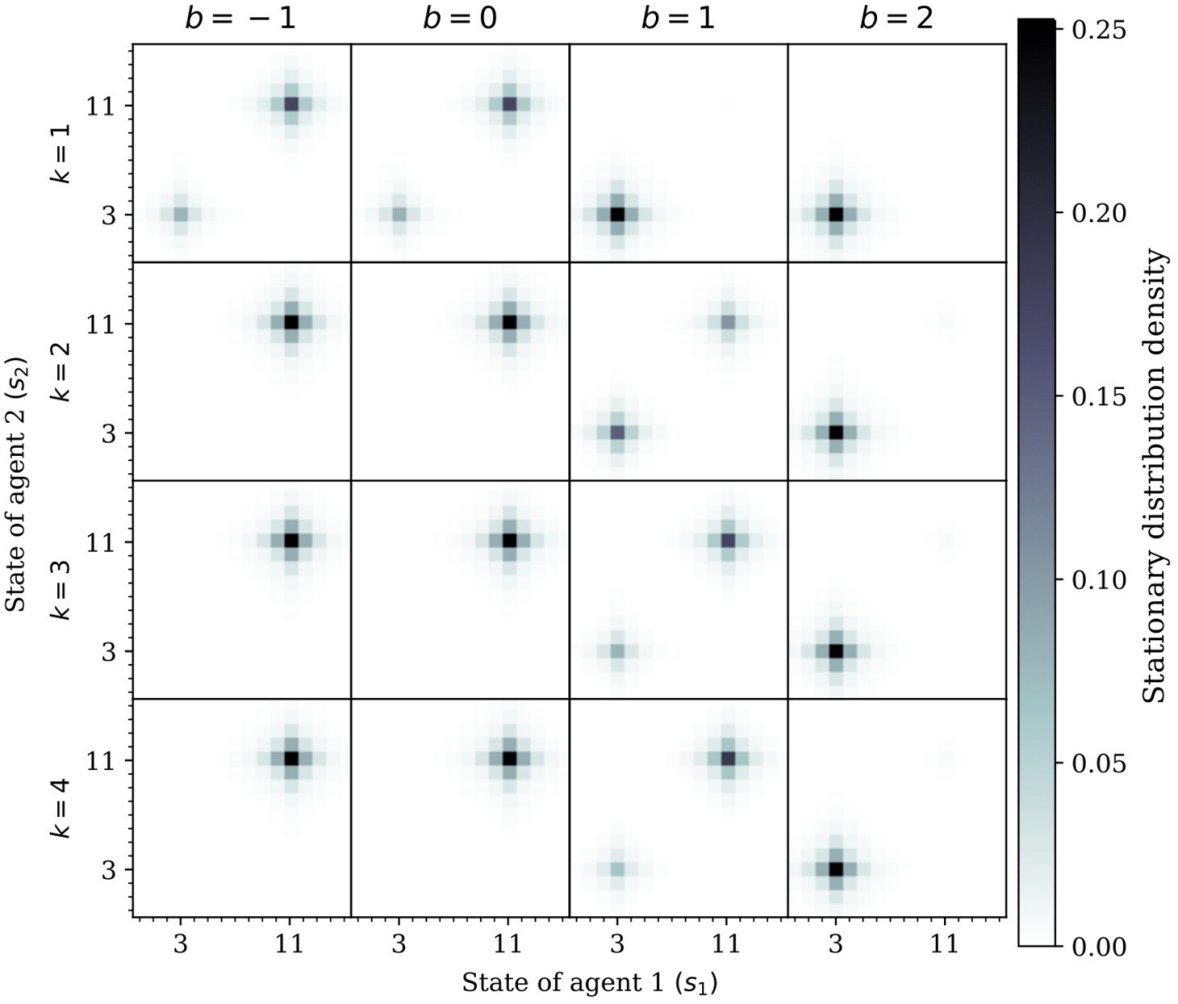
Role of the reference point of CPT-agents in the stationary distributions of the Markov stag hunt game for several sophistication levels. Stationary distributions of the resulting Markov chains obtained by conditioning the Markov game to increasingly sophisticated policies, *k* = 1, 2, 3 and 4, for CPT-agents with several reference points *b* = −1, 0, 1, 2. Darker colors indicate higher probability. We assumed discount factors *β*_1_ = *β*_2_ = 0.9, utility function *u*(*x*) = *x*, and weighting function $w\left(x\right)=e^{-0.5{\left(-\log\left(x\right)\right)}^{0.9}}$.

The reference point in cumulative prospect theory models the perception of losses and gains with respect to some predefined state of the agent which may be determined, in general, by its economical, social or psychological context. Agents with a high reference point tend to have a bleak perception of reality by perceiving most outcomes as losses, whereas agents with low reference point are optimistic and consider most outcomes as gains. In the case of the two hunters, evidence that theory of mind promotes coordination can be seen in the stationary distribution. Specifically, a high reference point steers hunters into the safety of hare hunting, whereas a low reference point steers them into the risky prospect of stag hunting. Since hunting stags is more uncertain—due to the need for both agents to coordinate their efforts—hunters with a high reference point will attempt to minimize their losses by playing it safe, a clear sign of CPT-induced risk-aversion. However, this effect is reversed when the reference point is below *b* = 1, where hunting stags is a more preferable option in the long-term—see Fig 8. Furthermore, the preference for hare hunting (i.e., the sub-optimal choice) can sometimes be overcome by more sophisticated theory of mind, since hunters can escape their risk sensitivity by changing their perception of what their partner is going to do. Specifically, at reference point *b* = 1, the equilibrium shifts from both agents preferring the safety of hares to both of them mostly hunting stags. This further suggests the importance of theory of mind in collective decision-making dilemmas.

## Discussion

### Theories of value

*Expected utility theory* (EUT) is likely the most adopted theory of value that provides a simple and parsimonious model to determine expected payoffs of uncertain outcomes but it is regarded as a prescriptive model of decision-making and is not viewed as a good descriptor of how humans assign value to uncertain outcomes [8]. In social situations, agents must make assumptions to be able to “predict” what the other agents will choose in order to be able to calculate the payoff of their actions. This axiomatic of rational choice [31] forms basis of EUT and has been observed not to be a very good descriptor of how people make decisions. The Allais paradox [32] and the Ellsberg paradox [33] are examples of how people break these axioms on a regular basis. In general settings, CPT offers a risk-sensitive generalization of EUT. In other words, EUT can be seen as particular cases of the framework proposed here, where the reference point is zero, and the utility functions (for gains and losses) and the probability weighting function are the identity.

CPT has been used to explain human behavior in many scenarios [34–42]. CPT is a powerful behavioral alternative to EUT, and empirical evidence suggests that CPT is a better model of human decision-making than EUT. Decision-making models achieved state of the art performance on human judgment datasets by creating neural networks with human-like inference bias by pre-training them with synthetic data generated by CPT [43].

### Theory of mind and risk sensitivity improve coordination

Our results suggest that CPT-agents, in a two-agent normal-form stag hunt game, can coordinate (hunt the stag) more effectively than EUT-agents using the simpler mixed Nash equilibrium. In other words, risk sensitivity can steer collective action, as hunting stag requires cooperation with the other individual in order to succeed [25]. This result, as expected, is sensitive to the way agents frame outcomes as either gains or losses, as well as how agents perceive the probability of success. Specifically, coordinated action is increased either when both hunters frame the game as a loss while overestimating low probabilities, or when hunters frame the game as a gain while underestimating high probabilities—see Fig 3.

Importantly, the conflict between short- and long-term rewards is a dilemma of particular interest in domains where time is a relevant factor in the most important collective problems humans face [44], from climate action to the impact of technology. Concurrently, it is also in these domains where a theory of mind may prove useful. We studied how agents coordinate in a Markov game version of two-player stag-hunt, where both agents were equipped with a level-*k* bounded rationality model, allowing them to predict several (increasingly sophisticated) behaviors in the form of policies.

In particular, our results provide evidence that increasingly sophisticated theory of mind promotes coordination in two-player games, but highly sophisticated theory of mind is not required for successful coordination, something that has been previously studied in evolutionary settings [45]. Our conclusions are valid for a wide range of parametric choices in our setting, namely in what concerns the reference point *b* (Fig 8) and the discount factor *β* (S3 Fig). Furthermore, when compared to the commonly used EUT-agents, CPT-agents coordinate faster, whereas the former fail to do so—see S4 Fig. In fact, we observe that even if only one hunter uses CPT while the other uses EUT to evaluate their actions, coordination is also increased compared to both hunters using EUT—see S5 Fig. These results show how risk sensitivity (using CPT) may help explain seemingly irrational but more realistic behaviors that cannot be explained using the more parsimonious EUT.

### Optimism increases coordination

Our results also suggest that hunters shift to hunting hares when the reference point is increased. It readily follows that using a higher reference point decreases the total reward. In other words, hunting stags is perceived as a not-so-good solution when both agents have a negative skewed view of the rewards; notice that CPT-agents with a higher reference point have a more bleak perception of rewards. However this effect can be mitigated (and sometimes nullified), since hunters may still switch to hunting stags if their sophistication level is high enough (cf. Fig 8 at *b* = 1). Additionally, higher sophistication levels (i.e., higher than *k* = 3) do not change the outcome of the two-agent setting in the long run. Thus, this provides further evidence that unbounded rationality is not only practically unfeasible, but also unnecessary for coordination. This suggests that the framing of gains and losses also plays an important role in the emergence of human coordination.

### Discounting future rewards decreases coordination

As mentioned in the Results section, the discount factor describes the hedonism of an agent and, as such, plays an important role in dynamic games. In our particular case, agent hedonism affects coordination and it is, therefore, worth studying. S3 Fig shows the stationary distribution of agents (EUT-agents and CPT-agents) for two different discount factors (i.e., *β* = 0.85 and *β* = 0.95), illustrating the effects of short-term versus long-term reward perception affect two-player coordination. Specifically, for *β* = 0.85, both agents prefer the hare state over the stag state, whereas for *β* = 0.95 the opposite is true and coordination is achieved. These results provide evidence that increasing the discount factor (i.e., increasing the perceived “goodness” of long-term rewards) also increases coordination of both EUT- and CPT-agents, and that the latter still generate more coordination than EUT. Furthermore, we observed that more sophisticated policies in the theory of mind help agents coordinate, even if the long-term reward consideration makes it unlikely at first.

### Diminishing marginal utility decreases coordination

S6 and S7 Figs show the equilibrium distributions when agents display a diminishing marginal utility when evaluating their actions. Specifically, agents use a concave utility function *u*(*x*) = *x*^λ^ with λ = 0.99 and λ = 0.95, respectively. These results suggest that a small change to the concavity of the utility function will render coordination difficult to attain, even with increased theory of mind sophistication. However, it was expected since the concave nature of the utility function makes smaller the difference between the utility of the stag state and that of the hare state, ergo making agents regard the stag state as less appealing relative to the hare state.

## Conclusion

Cognitive biases and theory of mind are a fundamental part of being human. We merged risk sensitivity and theory of mind into a novel theoretical framework and provided evidence that we are able to steer the behavior of individuals towards coordination among any two humans. Specifically, we studied the stag-hunt game in both its normal-form and Markov game versions and provided evidence that, by including cognitive biases and theory of mind in the dynamics of a two-player coordination game, agents are able to coordinate much more easily. Therefore, equipping agents with cumulative prospect theory helps coordination compared to the (standard) expected utility theory. This is a remarkable finding given that most multi-agent systems (MAS) use expected utility theory—likely due to the parsimonious mathematical framework it provides—and may be missing out on naturally occurring coordinating behaviors due to their focus on optimality. For example, in line with a recent work [46], prospect theory (a prequel of its cumulative counterpart studied here) has been used to study how fear of punishment can steer a population of individuals towards cooperation, even when punishment occurs very rarely. This is because the low probability of a very bad outcome is perceived (using prospect theory) to be more likely that what it is in reality, leading individuals toward more cooperative behaviors.

As shown here, behavioral agent models provide significantly different system dynamics compared to prescriptive agent models and, therefore, several interesting research directions naturally arise. For instance, multi-agent systems where agents represent people should use a descriptive behavioral model instead of a prescriptive model. Upon realizing this, one can start to develop and study human-based models such as idealized forms of democracy (e.g., liquid democracy [47]), video-game artificial intelligence with human-like behavior (or that is able to understand human-like behavior) and policy-making, or even revisiting already known social conflict problems such as the tragedy of the commons and the diffusion of responsibility.

It would also prove interesting to create an inference model to obtain the optimal parameters of this model, similar to [27] and [45]. For instance, a Bayesian method to infer the reference point, discount factor, utility and weighting function parameters, and policy sophistication level would enable machines to learn to act in a more personalized manner.

One caveat of obtaining bounded rationality using a level-*k* model is the assumption that stereotype policies are uniform, which may be rather unrealistic. Therefore, a way of creating more realistic stereotyped policies would be an interesting problem to tackle. One such way is self-play, a reinforcement learning method to train agents by pitting them against themselves and, in an evolutionary manner, preserving winners and discarding losers [48]. Furthermore, it is known that people represent their own mental states more distinctly than those of others [49], something to take into account when determining prior policies using the level-*k* bounded rationality model.

In the two-agent level-*k* model, it is known that humans, in general, do not use more sophistication than level-3 [50]. This creates a finite hypothesis space for the policy levels (i.e., with *k* = 0, 1, 2 and 3). However, when multiple interacting agents are a part of the environment, it is not enough to specify policy levels as a single number because each agent may have a policy which is a best response against several other policies of different levels. Therefore, there exists a problem of finding a behaviorally plausible hypothesis space for the inferred orders of each agent, which, if solved, would allow inference to be done on a collective level. Specifically, we would like reasoning such as “what you think about what he thinks that she thinks…” to be described in a simple, yet well-structured manner. This is crucial to understanding more general social dynamics because conclusions about two-player games do not often generalize to more players [51–54]. The team theory of mind model proposed in [55] is an interesting setting that tackles some of the problems but its solution is computationally costly.

Last but not least, verification of the proposed framework could be tested through behavioral experiments, which may also generate interesting data to further validate and expand the proposed model. These and other related research paths may lead to new models capable of capturing the the dynamics of systems comprised of people and, in turn, unlock the knowledge we lack to build artificial entities capable of understanding or fostering cooperation among humans and machines [13–17]. The present modeling approach can be further applied in the context of evolutionary models, potentially highlighting the impact and evolution of cognitive biases under the different classes of dilemmas humans faced throughout evolution.

## Methods

### CPT-value of a gamble with two outcomes

Cumulative prospect theory (CPT) provides a way to encode cognitive biases into the decision-making processes of cognitive agents. As we will see, these biases will significantly change the outcome of fairly simple decision scenarios. Suppose a CPT-agent is deciding between a certainty and gamble with two potential outcomes. The value of the certainty is straightforward, calculated as

$$\begin{array}{c} V^{Certainty}\left(b\right)=u\left(r-b\right), \end{array}$$ (7)

where *b* is the reference point, *r* is the certain outcome, and *u* is the following utility function:

$$\begin{array}{c} u\left(x\right)=\{\begin{array}{cc} x^{0.85} & \text{if}\;x\geq 0,\\ -{2|x|}^{0.85} & \text{if}\;x<0. \end{array} \end{array}$$ (8)

The value of the gamble with two outcomes, *r*_+_ with probability *p* and *r*_−_ with probability 1 − *p* (*r*_+_ > *r*_−_) is calculated as follows:

$$\begin{array}{c} V^{Gamble}\left(b,\alpha \right)=u\left(r_{+}-b\right)\psi \left(r_{+}-b\right)+u\left(r_{-}-b\right)\psi \left(r_{-}-b\right), \end{array}$$ (9)

where *α* is the Prelec parameter, *r*_+_ (*r*_−_) is the highest (lowest) outcome, and *ψ*(*x*) is the perceived likelihood of outcome *x* computed as follows:

$$\begin{array}{c} \begin{array}{cc} \psi (r_{+}-b) & =\{\begin{array}{cc} w(p) & \text{if}\;r_{+}-b\geq 0,\\ 1-w(1-p) & \text{otherwise,}\; \end{array}\\ \psi (r_{-}-b) & =\{\begin{array}{cc} 1-w(p) & \text{if}\;r_{-}-b\geq 0,\\ w(1-p) & \text{otherwise,}\; \end{array} \end{array} \end{array}$$ (10)

where *w*(*p*) = exp{−*α*(−log(*p*))^*δ*^} is Prelec’s probability weighting function [24]. A more general formulation is provided to the reader in Section 1 of S1 Text.

### CPT-value in symmetric 2-player 2-actions normal-form games

In a symmetric normal-form game with two players (1 and 2) and two actions—cooperate (C) and defect (D)—the payoff matrix can be written as in Table 2.

**Table 2:** Payoff matrix of a symmetric 2-player, 2-action normal-form game.

		Player 2	
		*C*	*D*
Player 1	*C*	*R*, *R*	*S*, *T*
	*D*	*T*, *S*	*P*, *P*

A Nash equilibrium prescribes the situation where each player acts to make the other player’s decisions have equal value. Therefore, player 1 calculates the value of player 2’s actions, as follows:

$$\begin{array}{c} \begin{array}{cc} V_{2}\left(C\right) & =u(R-b)\psi (R-b)+u(S-b)\psi (S-b),\\ V_{2}\left(D\right) & =u(T-b)\psi (T-b)+u(P-b)\psi (P-b),\\ V_{2}\left(C\right) & =V_{2}\left(D\right), \end{array} \end{array}$$ (11)

where the likelihoods *ψ* are calculated as in CPT. In other words, player 1 will then rewrite the last equality of Eq 11 to obtain the probability of his actions which make player 2 indifferent between his actions. Since the game is symmetric, player 2 will have the same policy. A brief introduction to normal-form games and its CPT formulation can be found in Section 2 of S1 Text.

### Infinite horizon CPT-value in Markov games

A Markov game is a multiplayer generalization of a Markov Decision Process (MDP). Section 3 of S1 Text provides a brief introduction to MDPs and Markov games in the context of CPT, as well as how agents decide in the context of theory of mind applied to Markov games. Briefly, the CPT-value that agent *i* places on a joint state $(s_{1},...,s_{n})=s\in S$, given a joint policy **π** = (*π*_*i*_, **π**_−*i*_), can be obtained by generalizing the MDP CPT-value to the Markov game via successive iterations of

$$\begin{array}{c} \begin{array}{cc} V_{i}^{\pi_{i},\pi_{-i}}\left(s\right) & =\int_{0}^{\infty }w_{i}^{+}(\sum_{a_{i}\in A_{i}}P_{i,s,+}^{a_{i},\pi_{-i}}\left(\epsilon \right)\pi_{i}\left(a_{i}|s\right))d\epsilon \\ & -\int_{0}^{\infty }w_{i}^{-}(\sum_{a_{i}\in A_{i}}P_{i,s,-}^{a_{i},\pi_{-i}}\left(\epsilon \right)\pi_{i}\left(a_{i}|s\right))d\epsilon , \end{array} \end{array}$$ (12)

where

$$\begin{array}{c} \begin{array}{cc} & P_{i,s,+}^{a_{i},\pi_{-i}}\left(\epsilon \right)=\sum_{a_{-i}\in A_{-i}\left(s\right)}P_{i,s,+}^{a_{i},a_{-i}}\left(\epsilon \right)\pi_{-i}\left(a_{-i}|s\right),\\ & P_{i,s,-}^{a_{i},\pi_{-i}}\left(\epsilon \right)=\sum_{a_{-i}\in A_{-i}\left(s\right)}P_{i,s,-}^{a_{i},a_{-i}}\left(\epsilon \right)\pi_{-i}\left(a_{-i}|s\right), \end{array} \end{array}$$

$$\begin{array}{c} \begin{array}{cc} P_{i,s,+}^{a_{i},a_{-i}}\left(\epsilon \right) & =P_{s}^{a}\left(u_{i}^{+}\left({\left(r_{i}\left(s\right)+\beta_{i}V_{i}^{\pi_{i},\pi_{-i}}\left(S\right)-b_{i}\right)}_{+}\right)>\epsilon \right),\;\text{and}\;\\ P_{i,s,-}^{a_{i},a_{-i}}\left(\epsilon \right) & =P_{s}^{a}\left(u_{i}^{-}\left({\left(r_{i}\left(s\right)+\beta_{i}V_{i}^{\pi_{i},\pi_{-i}}\left(S\right)-b_{i}\right)}_{-}\right)>\epsilon \right). \end{array} \end{array}$$

Each agent *i* tries to maximize his value *V*_*i*_ by choosing the optimal policy *π*_*i*_ given the joint policy of every other agent **π**_−*i*_, i.e.,

$$\begin{array}{c} \pi_{i}^{*}\left(s\right)=\underset{\pi_{i}}{\text{argmax}}\;V_{i}^{\pi_{i},\pi_{-i}}\left(s\right),\forall s\in S. \end{array}$$ (13)

### Creating simultaneous markov games from individual Markov decisions processes

To create a simultaneous decision-making scenario, the dynamics in the joint state space and the individual transition probabilities must be combined in a proper manner, i.e.,

$$\begin{array}{c} P^{a_{1},a_{2}}=\frac{I⊗P^{a_{1}}+P^{a_{2}}⊗I}{2}, \end{array}$$ (14)

where the Kronecker product ⊗ ensures an action from agent 1 does not change the state of agent 2 and vice-versa. The average of this transformed agent transition probability function ensures the joint state space dynamics is independent of who acts first.

### Stationary distribution

Agent behavior can be summarized by the stationary distribution of a Markov game, when this is conditioned on a joint policy—effectively turning a Markov game into a Markov chain. This stationary distribution conveys information about the joint states the two-agent system will most likely be in (in the long-run).

When agent 1 uses policy $\pi_{1}^{\left(k_{1}\right)}$ and agent 2 uses policy $\pi_{2}^{\left(k_{2}\right)}$, the resulting conditioned transition probability function can be obtained as

$$\begin{array}{c} P_{s,s^{\prime }}^{\pi_{1},\pi_{2}}=\sum_{a_{1},a_{2}}P_{s,s^{\prime }}^{a_{1},a_{2}}\pi_{1}\left(a_{1}|s\right)\pi_{2}\left(a_{2}|s\right). \end{array}$$

From this, the stationary distribution *ρ*(*k*) when agents use $\pi_{1}^{\left(k\right)}$ and $\pi_{2}^{\left(k\right)}$—that is, the same policy at *k*-level of bounded rationality—can be obtained via

$$\begin{array}{c} \rho \left(k\right)=\rho \left(k\right)P^{\pi_{1}^{\left(k\right)},\pi_{2}^{\left(k\right)}}. \end{array}$$

### Limitations

Prospect theory and its cumulative extension propose an original probability weighting function [8, 9] which is different from the Prelec probability weighting function [24] used here—see Fig 1 for a comparison between the two. We have considered the Prelec probability weighting function for two main reasons. First, it generalizes the original probability weighting function used in the CPT context such that both the certainty and possibility effects are still present, while allowing us to control (by changing *α*) which of these two effects dominated probability perception. Second, when computing the CPT value of the Markov game, it leads to highly unstable numerical issues that ultimately results in failure to converge for a wide range of constrained non-linear optimization methods. This, however, is an effect that persisted even when attempting to approximate Prelec’s probability weighting function—see details in Section 4 of S1 Text.

## Supporting information

The various sections of S1 Text provide detailed information about the proposed model. In Section 1 of S1 Text, we show how agents determine value of outcomes—or, equivalently, actions—using *expected utility theory* (EUT) and *cumulative prospect theory* (CPT). This is followed by a brief introduction to normal-form games, in Section 2 of S1 Text, and Markov games, with a technical overview of how to calculate CPT-value in Markov games and how the level-*k* model plays a role in this calculation, in Section 3 of S1 Text. Lastly, in Section 4 of S1 Text, we discuss some limitations of this model.

S1 Text Detailed information about the proposed model. (PDF)

S1 FigEUT and CPT values as functions of the agent states, for sophistication levels *k* = 1, 2, 3, 4.Joint states with redder colors have higher value. We assumed reference points *b*_1_ = *b*_2_ = 0, discount factors *β*_1_ = *β*_2_ = 0.9, utility function *u*(*x*) = *x* and weighting function *w*(*x*) = *x* for EUT, and $w\left(x\right)=e^{-0.5{\left(-\log\left(x\right)\right)}^{0.9}}$ for CPT.(TIF)

S2 FigStationary distributions of the resulting Markov chains obtained by conditioning the Markov game to increasingly sophisticated policies (*k* = 1, 2, 3, 4) for EUT- and CPT-agents.Joint states with darker color indicate larger probability. We assumed reference points *b*_1_ = *b*_2_ = 0, discount factors *β*_1_ = *β*_2_ = 0.9, utility function *u*(*x*) = *x*, and weighting function *w*(*x*) = *x* for EUT, and $w\left(x\right)=e^{-0.5{\left(-\log\left(x\right)\right)}^{0.9}}$ for CPT.(TIF)

S3 FigRole of discount factor *β* on the stationary distributions of the resulting Markov chains obtained by conditioning the Markov game to increasingly sophisticated policies (*k* = 1, 2, 3, 4) for EUT-agents and CPT-agents.Joint states with darker color indicate larger probability. We assumed reference points *b*_1_ = *b*_2_ = 0, utility function *u*(*x*) = *x* and weighting function *w*(*x*) = *x* for EUT and $w\left(x\right)=e^{-0.5{\left(-\log\left(x\right)\right)}^{0.9}}$ for CPT. (Left) Stationary distribution for EUT- and CPT-agents using discount factor *β* = 0.85. (Right) Stationary distribution for EUT- and CPT-agents using discount factor *β* = 0.95.(TIF)

S4 Fig Role of sophistication level of two EUT-agents on the stationary distributions of the Markov stag hunt game. Joint states with darker color indicate larger probability. We assumed equal agent parameters: discount factors *β*_1_ = *β*_2_ = 0.9, reference point *b*_1_ = *b*_2_ = 0, utility functions *u*_1_(*x*) = *u*_2_(*x*) = *x*, and weighting function *w*_1_(*x*) = *w*_2_(*x*) = *x*).
(TIF)

S5 FigRole of sophistication level of one EUT-agent (agent 1) and one CPT-agent (agent 2) on the stationary distributions of the Markov stag hunt game.Joint states with darker color indicate larger probability. Here, agent parameters are fixed at: discount factors *β*_1_ = *β*_2_ = 0.9, reference point *b*_1_ = *b*_2_ = 0, utility functions *u*_1_(*x*) = *u*_2_(*x*) = *x*, and weighting functions *w*_1_(*x*) = *x*, and $w_{2}\left(x\right)=e^{-0.5{\left(-\log\left(x\right)\right)}^{0.9}}$.(TIF)

S6 FigStationary distributions of the Markov stag hunt game for asymmetric sophistication levels of CPT-agents with utility function concavity parameter at 0.99.Joint states with darker color indicate larger probability. We assumed equal agent parameters (i.e., discount factors *β*_1_ = *β*_2_ = 0.9, reference point *b*_1_ = *b*_2_ = 0, utility functions *u*_1_(*x*) = *u*_2_(*x*) = *x*^0.99^, and weighting function $w_{1}\left(x\right)=w_{2}\left(x\right)=e^{-0.5{\left(-\log\left(x\right)\right)}^{0.9}}$).(TIF)

S7 FigStationary distributions of the Markov stag hunt game for asymmetric sophistication levels of CPT-agents with utility function concavity parameter at 0.95.Joint states with darker color indicate larger probability. We assumed equal agent parameters (i.e., discount factors *β*_1_ = *β*_2_ = 0.9, reference point *b*_1_ = *b*_2_ = 0, utility functions *u*_1_(*x*) = *u*_2_(*x*) = *x*^0.95^, and weighting function $w_{1}\left(x\right)=w_{2}\left(x\right)=e^{-0.5{\left(-\log\left(x\right)\right)}^{0.9}}$).(TIF)
