# Supplementary material for: Risk sensitivity and theory of mind in human coordination
Source: PLoS Comput Biol. 2021 Jul 15;17(7):e1009167. doi: 10.1371/journal.pcbi.1009167 (PMC8315544; doi:10.1371/journal.pcbi.1009167)
Supplement: S1 Text — (PDF) [file pcbi.1009167.s001.pdf]

S1 Text of "Risk sensitivity and theory of mind in human coordination"

Pedro L. Ferreira[1], Francisco C. Santos[1], Sérgio Pequito[2*]

1 INESC-ID and Instituto Superior Técnico, Universidade de Lisboa, Lisboa, Portugal
2 Center for Systems and Control, Delft University of Technology, Delft, Netherlands

*sergio.pequito@tudelft.nl

In what follows we provide detailed information about the proposed model. In Section 1, we show how agents determine value of outcomes – or, equivalently, actions – using *expected utility theory* (EUT) and *cumulative prospect theory* (CPT). This is followed by a brief introduction to normal-form games, in Section 2, and Markov games, with a technical overview of how to calculate CPT-value in Markov games and how the level-k model plays a role in this calculation, in Section 3. Lastly, in Section 4, we discuss some limitations of this model.

1 Determining the value of outcomes

The determination of value is often done using *expected utility theory* (EUT). Let R be a discrete random variable with distribution over the set of outcomes $\{r_j\}_{j=1}^m$ (sorted in increasing order), and $p_j = \mathbb{P}(R = r_j)$ be the probability that outcome r_j occurs. Let also $u : \mathbb{R} \to \mathbb{R}$ be a utility function that describes how an agent perceives rewards. The value of R (sometimes referred to as a *lottery* or *prospect* in the literature) under EUT is the expected value of its utility given by

$$V^{\text{EUT}}(R) = \sum_{j=1}^{m} u(r_j)p_j. \tag{1}$$

Taking u to be the identity function, EUT is simply the mathematical expectation of the rewards.

To determine the value under CPT, let us first define the following auxiliary functions:

$$\psi^+(r_j) = w^+(P(R \geq r_j)) - w^+(P(R > r_j)), \text{ and}$$
$$\psi^-(r_j) = w^-(P(R \leq r_j)) - w^-(P(R < r_j)),$$

where $w^+ : [0,1] \to [0,1]$ and $w^- : [0,1] \to [0,1]$ are probability weighting functions for gains and losses, respectively. In contrast, the value of R under CPT is given by

$$V^{\text{CPT}}(R) = \sum_{j \geq k} u^+(r_j)\psi^+(r_j) + \sum_{j<k} u^-(r_j)\psi^-(r_j), \tag{2}$$

where $k \in \mathbb{Z}$ is the reference point, which splits the prospect into gains and losses, and u^+ and u^- are the utility functions for gains and losses, respectively. CPT is a generalization of EUT in the sense that $V^{\text{CPT}} = V^{\text{EUT}}$, if $u^+(x) = u^-(x) = u(x)$, for all $x \in \mathbb{R}$ and $w^+(p) = w^-(p) = p$, for all $p \in [0,1]$.

2 The normal-form game

A normal-form game is composed of a set of agents (or players) $N = \{1, ..., n\}$ and, for each agent i, a set of actions A_i and a utility function $u_i : A_1 \times ... \times A_n \to \mathbb{R}$. A play of a normal-form game consists in all agents simultaneously (or, equivalently, without knowing the others' chosen actions) choosing an action from their respective action sets and receiving their respective utilities based on the chosen actions.

The behavior of each agent i is represented as a policy denoted by π_i ($\pi_i : A_i \to [0, 1]$ and $\sum_{a \in A_i} \pi_i(a) = 1$). For instance, if agent 1 decides to choose an action a_1 (out of two possible actions, i.e., $\{a_1, a_2\}$) with probability 0.2, then his policy is $\pi_1(a_1) = 0.2$ and $\pi_1(a_2) = 0.8$. A joint policy $\boldsymbol{\pi}$ is the collection of the chosen policies of all agents. In general, to solve a normal-form game one needs to find the Nash equilibria (NEs) (i.e., joint policies where no agent will receive a higher payoff by unilaterally changing its action).

Mixed Nash Equilibrium. The mixed Nash equilibria of a game is the subset of all Nash equilibria in which agents are allowed to play each action with some probability, rather than playing deterministically. Since the Nash equilibrium is a joint policy which yields no better payoff by any agent switching, that implies that, in a game with two agents, each agent will choose the policy that makes the other agent value their actions equally. Mathematically, agent 1 will choose π_1 that makes the value of each action equal, i.e.,

$$V_2(a_1|\pi_1) = V_2(a_2|\pi_1) = ... = V_2(a_{|A_2|}|\pi_1), \forall a_i \in A_2,$$

and the same happens for agent 2 when determining π_2.

The canonical symmetric 2-player normal-form game with two actions, cooperate (C) and defect (D), can be represented in the following payoff matrix

		Agent 2 C	Agent 2 D
Agent 1	C	R, R	S, T
	D	T, S	P, P

where R is the *reward*, S is the *sucker's payoff*, T is the *temptation*, and P is the *punishment*. In this case, the values of each action can be computed in the standard expected value theory as:

$$V(C|p) = Rp + S(1-p),$$

$$V(D|p) = Tp + P(1-p),$$

where p is the probability either agent chooses to cooperate (i.e., action C). The mixed Nash equilibrium can be computed by equating the above expressions, yielding

$$p_{Nash} = \frac{P - S}{R - T + P - S}.$$

Of course, the above expression for the mixed Nash equilibrium is only valid if the division is possible and yields a probability (i.e., $0 \le p_{Nash} \le 1$).

Under CPT, the values of each action are

$$V^{CPT}(C|p,b,\alpha) = u(R-b)w(p|\alpha) + u(S-b)w(1-p|\alpha), \text{ and}$$
$$V^{CPT}(D|p,b,\alpha) = u(T-b)w(p|\alpha) + u(P-b)w(1-p|\alpha),$$

where we made explicit that b is the reference point of the agents and α is a parameter in the probability weighting function. We are assuming it is the same for both agents, but this is done for simplicity; in other words, one can assume different values for the reference point, as well as different utility functions u and probability weighting functions w and study the equilibria that follow from that asymmetry.

The equilibrium policy under CPT, denoted by p_{CPT}, can be obtained by finding the policy that equates the CPT-value of both actions, i.e.,

$$V^{CPT}(C|p_{CPT},b,\alpha) = V^{CPT}(D|p_{CPT},b,\alpha).$$

3 The Markov game

Repeated games allow for the study of the interaction between immediate gains and long-term incentives. We provide a brief review of the *Markov decision process* (MDP) – a single-agent decision model – and explain how they relate to the Markov game – a model of collective decision-making.

A MDP is a model of discrete-time decision-making in a stochastic environment. Formally, it consists of a set of states $\mathcal{S}$, a set of actions $\mathcal{A}$, a probability transition function $p : \mathcal{S}\times\mathcal{A}\times\mathcal{S} \to [0,1]$, and a reward function $r : \mathcal{S}\times\mathcal{A}\times\mathcal{S} \to \mathbb{R}$. In general, in this framework, an agent is attempting to find a policy $\pi : \mathcal{S}\times\mathcal{A} \to [0,1]$, that maximizes some value functional $V(s,\pi)$, for all states $s \in \mathcal{S}$.

To determine the policies of a MDP, for the standard infinite-horizon EUT value functional, we can use dynamic programming. This technique makes use of a discount factor $\beta \in (0,1)$ that accounts for the importance of short-term versus long-term rewards. Similarly, dynamic programming can be used when the infinite-horizon CPT value functional is considered [1, 2].

A Markov game is a generalization of a MDP to accommodate several agents [3]. The main difference is that, in Markov games, each agent must take into account the possible policies of other agents whilst maximizing his own payoff at each state. Formally, the number of agents $n > 1$ in a Markov game require a joint action space $\boldsymbol{\mathcal{A}} = \mathcal{A}_1 \times ... \times \mathcal{A}_n$ (where $\mathcal{A}_i$ is the action space of agent i and $\mathcal{A}_{-i}$ is the joint action space of all agents except agent i), and a joint state space $\boldsymbol{\mathcal{S}} = \mathcal{S}_1 \times ... \times \mathcal{S}_n$ (where $\mathcal{S}_i$ is the state space of agent i), with stochastic dynamics prescribed by the transition function $P_{\boldsymbol{s}}^{\boldsymbol{a}}(\cdot) = \mathbb{P}(\cdot|\boldsymbol{s}_t = \boldsymbol{s}, \boldsymbol{a}_t = \boldsymbol{a})$, $(s_1,...,s_n) = \boldsymbol{s} \in \boldsymbol{\mathcal{S}}$, $(a_1,...,a_n) = \boldsymbol{a} \in \boldsymbol{\mathcal{A}}$.

The CPT-value that agent i places on a joint state $(s_1,...,s_n) = \boldsymbol{s} \in \boldsymbol{\mathcal{S}}$, given a joint policy $\boldsymbol{\pi} = (\pi_i, \boldsymbol{\pi}_{-i})$, can be obtained by generalizing the MDP CPT-value [1] to the Markov game via successive iterations of

$$\begin{aligned} V_i^{\pi_i,\boldsymbol{\pi}_{-i}}(\boldsymbol{s}) = &\int_0^\infty w_i^+ \left(\sum_{a_i \in \mathcal{A}_i} P_{i,\boldsymbol{s},+}^{a_i,\boldsymbol{\pi}_{-i}}(\epsilon)\pi_i(a_i|\boldsymbol{s}) \right) d\epsilon \\ &- \int_0^\infty w_i^- \left(\sum_{a_i \in \mathcal{A}_i} P_{i,\boldsymbol{s},-}^{a_i,\boldsymbol{\pi}_{-i}}(\epsilon)\pi_i(a_i|\boldsymbol{s}) \right) d\epsilon, \end{aligned} \tag{3}$$

where

$$P_{i,\boldsymbol{s},+}^{a_i,\boldsymbol{\pi}_{-i}}(\epsilon) = \sum_{\boldsymbol{a}_{-i}\in\boldsymbol{\mathcal{A}}_{-i}(\boldsymbol{s})} P_{i,\boldsymbol{s},+}^{a_i,\boldsymbol{a}_{-i}}(\epsilon)\boldsymbol{\pi}_{-i}(\boldsymbol{a}_{-i}|\boldsymbol{s}),$$
$$P_{i,\boldsymbol{s},-}^{a_i,\boldsymbol{\pi}_{-i}}(\epsilon) = \sum_{\boldsymbol{a}_{-i}\in\boldsymbol{\mathcal{A}}_{-i}(\boldsymbol{s})} P_{i,\boldsymbol{s},-}^{a_i,\boldsymbol{a}_{-i}}(\epsilon)\boldsymbol{\pi}_{-i}(\boldsymbol{a}_{-i}|\boldsymbol{s}),$$

$$P_{i,\boldsymbol{s},+}^{a_i,\boldsymbol{a}_{-i}}(\epsilon) = P_{\boldsymbol{s}}^{\boldsymbol{a}}(u_i^+((r_i(\boldsymbol{s}) + \beta_i V_i^{\pi_i,\boldsymbol{\pi}_{-i}}(\boldsymbol{S}) - b_i)_+) > \epsilon), \text{ and}$$
$$P_{i,\boldsymbol{s},-}^{a_i,\boldsymbol{a}_{-i}}(\epsilon) = P_{\boldsymbol{s}}^{\boldsymbol{a}}(u_i^-((r_i(\boldsymbol{s}) + \beta_i V_i^{\pi_i,\boldsymbol{\pi}_{-i}}(\boldsymbol{S}) - b_i)_-) > \epsilon).$$

Each agent i tries to maximize his value V_i by choosing the optimal policy π_i given the joint policy of every other agent $\boldsymbol{\pi}_{-i}$, i.e.,

$$\pi_i^*(\boldsymbol{s}) = \operatorname*{argmax}_{\pi_i} V_i^{\pi_i,\boldsymbol{\pi}_{-i}}(\boldsymbol{s}), \forall \boldsymbol{s} \in \boldsymbol{\mathcal{S}}. \tag{4}$$

The level-k bounded rationality model. To obtain the solution to (4), agent i requires knowledge of $\boldsymbol{\pi}_{-i}$. Instead of assuming rational agents, we will consider that agents have bounded rationality according to the level-k bounded rationality model. In a two-agent setting, agent 1 assumes a stereotyped policy $\pi_2^{(0)}$, which describes the behavior of an independently operating agent 2. Similarly, agent 2 assumes a stereotyped policy $\pi_1^{(0)}$, which describes the behavior of an independently operating agent 1. With this "new" information, both agents can determine their first-order policies $\pi_1^{(1)}$ and $\pi_2^{(1)}$. However, each of them can also assume that the other did the same and is, therefore, operating under a first-order policy. Subsequently, they should operate under a second-order policy, and so forth. Thus, if such reasoning is used k times, we obtain the following recursive scheme (each line is an iteration and the left and right hand sides represent agent 1 and 2 decisions, respectively):

$$\begin{array}{rcl} & \pi_2^{(0)} \quad \pi_1^{(0)} & \\ & \downarrow \quad\;\; \downarrow & \\ \operatorname*{argmax}_{\pi_1} V_1(\pi_1, \pi_2^{(0)}) = \pi_1^{(1)} & & \pi_2^{(1)} = \operatorname*{argmax}_{\pi_2} V_2(\pi_1^{(0)}, \pi_2), \\ \vdots & & \vdots \\ \operatorname*{argmax}_{\pi_1} V_1(\pi_1, \pi_2^{(k-1)}) = \pi_1^{(k)} & & \pi_2^{(k)} = \operatorname*{argmax}_{\pi_2} V_2(\pi_1^{(k-1)}, \pi_2). \end{array} \tag{5}$$

4 Limitations

The solution to the optimization problem defined in Equation 4 is often a daunting task as the numerical approaches suffer from (well known) instability issues for some initial configurations. Consequently, when this occurred, a different but valid initial configuration was selected at random until convergence.

Additionally, the weighting function $w(x) = e^{-0.5(-\log(x))^{0.9}}$ is both computationally expensive and its implementation has to be truncated as $x \to 0$. Therefore, a posynomial approximation

$w(x) = 0.00231x^{0.05}+0.00128x^{0.1}+0.19578x^{0.35}+0.59897x^{0.4}+0.15968x^{0.95}+0.03318x^{3}+0.00847x^{23}$ was used, similar to [4].

The reader should also be made aware that the optimization algorithm itself is slow and relatively unstable to some parameter configurations, particularly for convex probability weighting functions and high discount factors. Theoretical work on techniques regarding CPT value optimization would prove useful and allow us to readily study agent-based systems with more than two agents and at more extreme parameter configurations.

References

1. Lin K, Marcus SI. Dynamic Programming with Non-Convex Risk-Sensitive Measures. In: Proceedings of the American Control Conference; 2013. p. 6778–6783.

2. Lin K. Stochastic Systems with Cumulative Prospect Theory [PhD dissertation]. University of Maryland, College Park; 2013.

3. Shapley LS. Stochastic Games. In: Proceedings of the National Academy of Sciences; 1953. p. 1095–1100.

4. Cubuktepe M, Topcu U. Verification of Markov Decision Processes with Risk-Sensitive Measures. In: Proceedings of the American Control Conference. IEEE; 2018. p. 2371–2377.
