# Supplementary figures and images for: Risk sensitivity and theory of mind in human coordination
Source: PLoS Comput Biol. 2021 Jul 15;17(7):e1009167. doi: 10.1371/journal.pcbi.1009167 (PMC8315544; doi:10.1371/journal.pcbi.1009167)

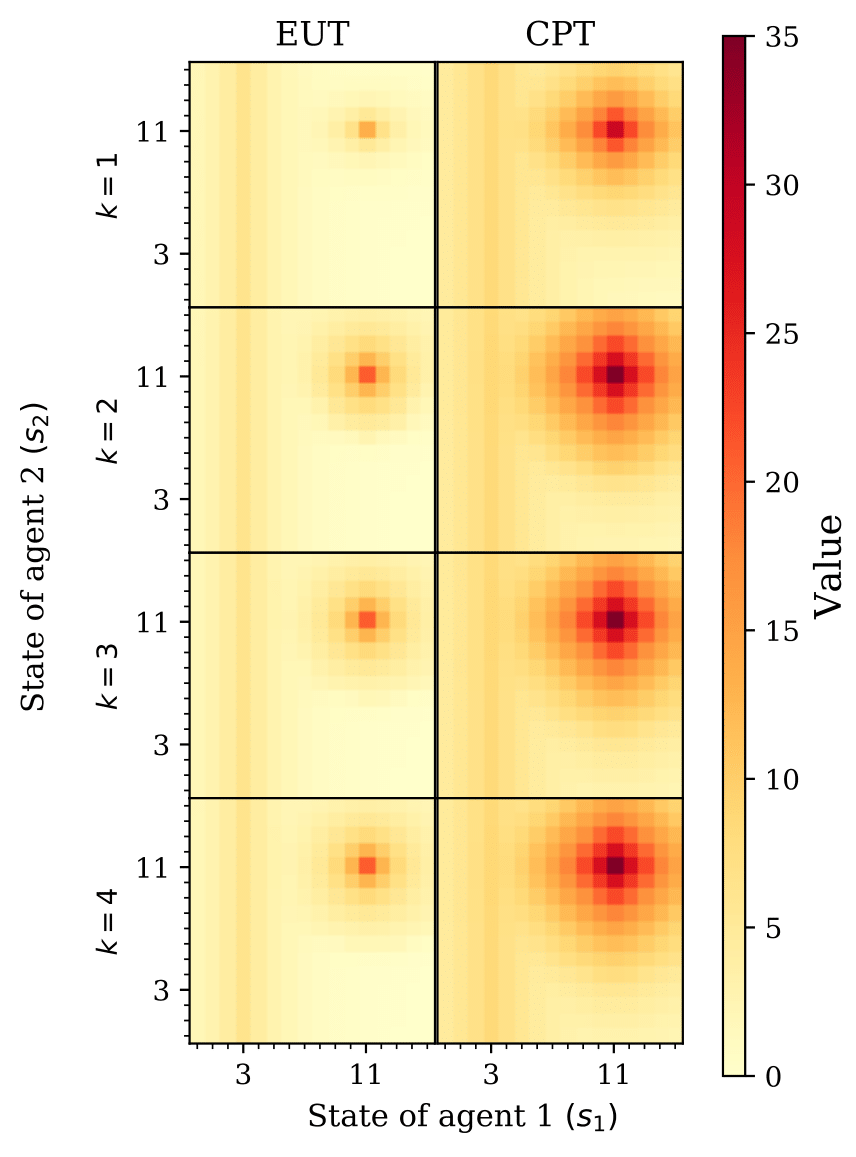

Supplement: S1 Fig — Joint states with redder colors have higher value. We assumed reference points b1 = b2 = 0, discount factors β1 = β2 = 0.9, utility function u(x) = x and weighting function w(x) = x for EUT, and w(x)=e-0.5(-log(x))0.9 for CPT. (TIF) [file pcbi.1009167.s002.tif]

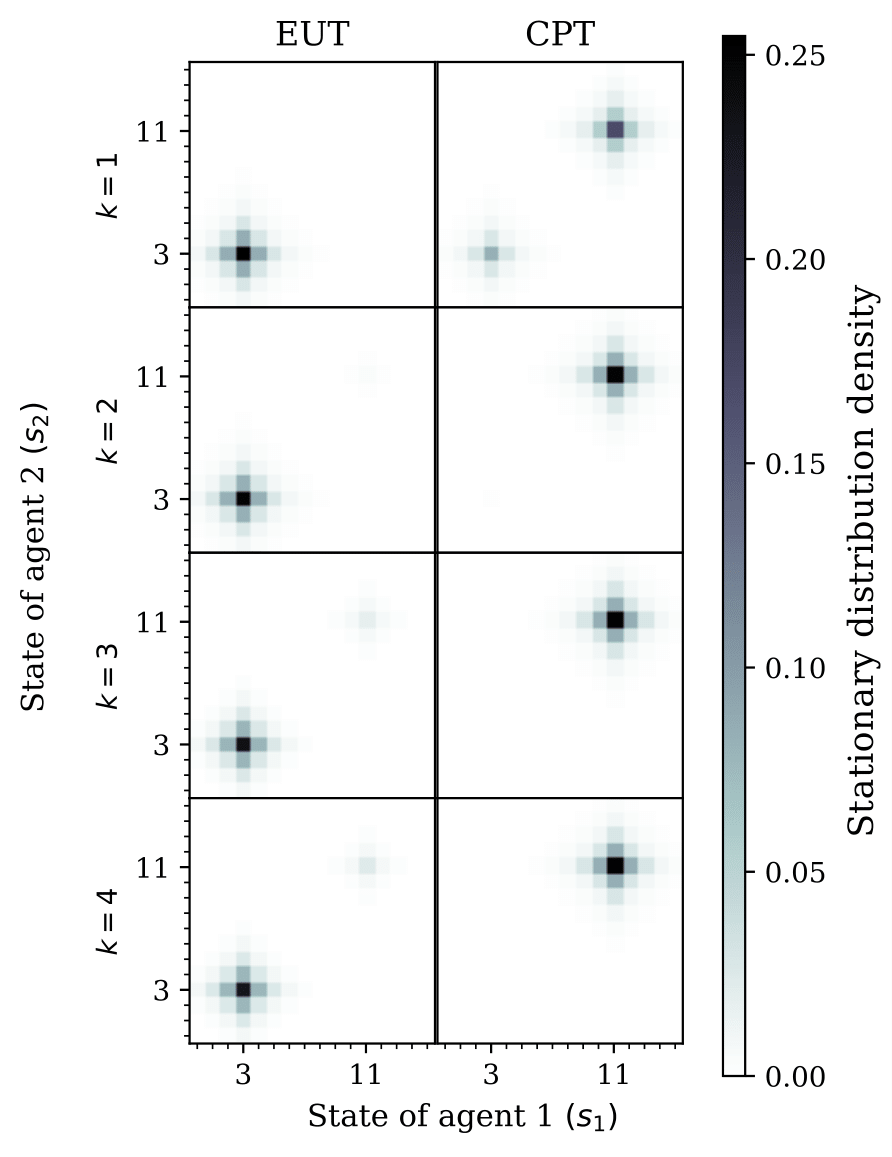

Supplement: S2 Fig — Joint states with darker color indicate larger probability. We assumed reference points b1 = b2 = 0, discount factors β1 = β2 = 0.9, utility function u(x) = x, and weighting function w(x) = x for EUT, and w(x)=e-0.5(-log(x))0.9 for CPT. (TIF) [file pcbi.1009167.s003.tif]

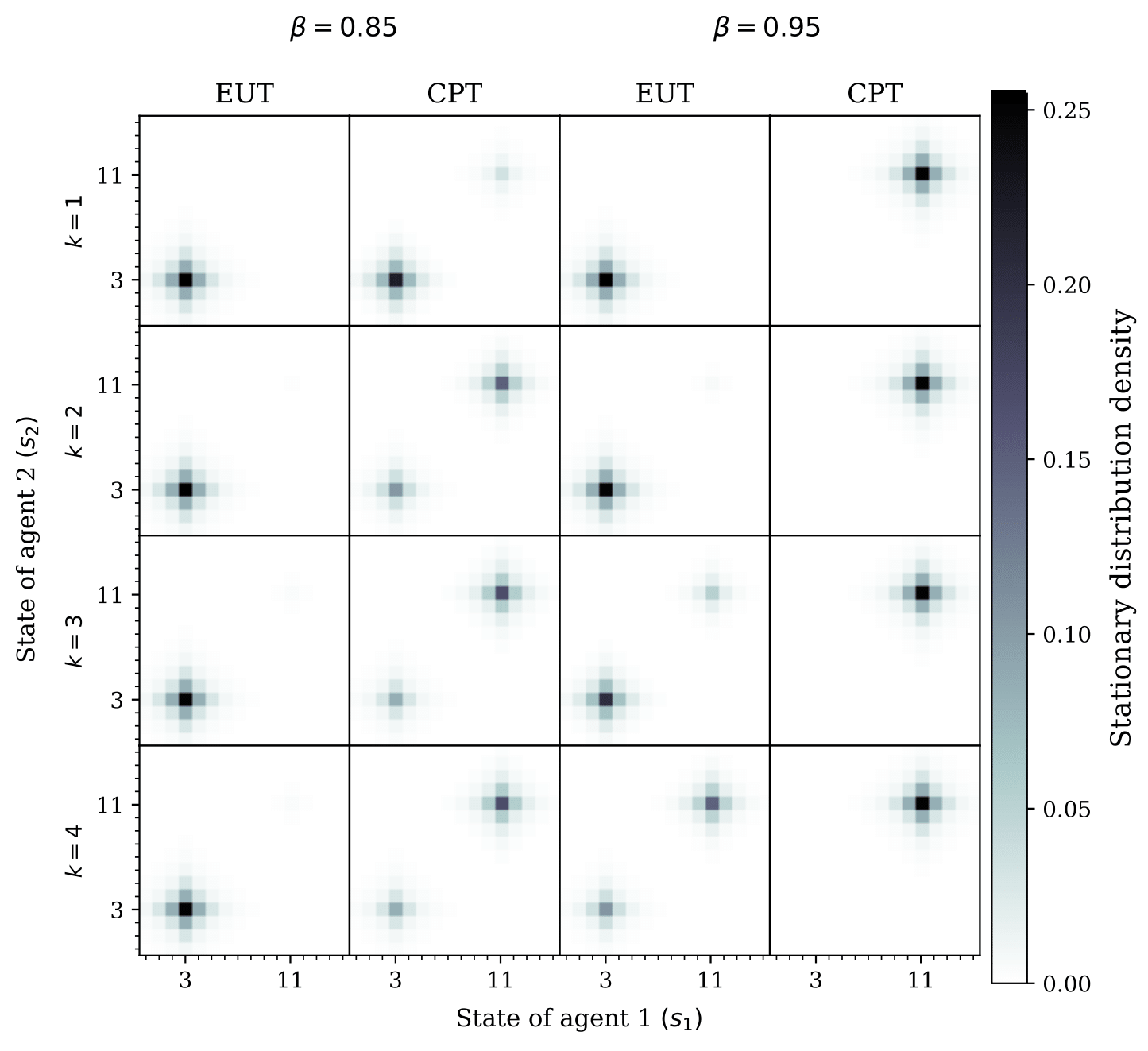

Supplement: S3 Fig — Joint states with darker color indicate larger probability. We assumed reference points b1 = b2 = 0, utility function u(x) = x and weighting function w(x) = x for EUT and w(x)=e-0.5(-log(x))0.9 for CPT. (Left) Stationary distribution for EUT- and CPT-agents using discount factor β = 0.85. (Right) Stationary distribution for EUT- and CPT-agents using discount factor β = 0.95. (TIF) [file pcbi.1009167.s004.tif]

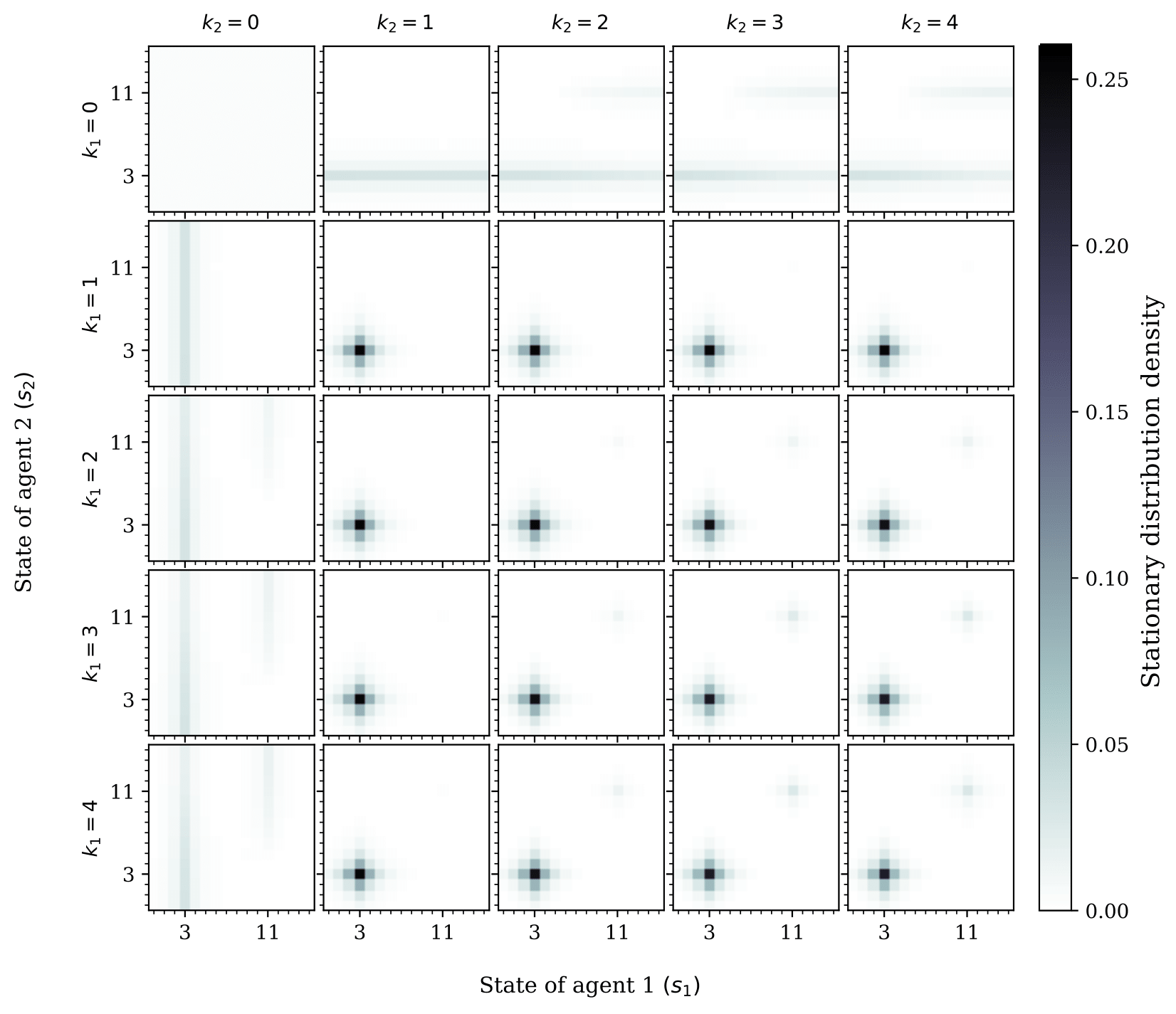

Supplement: S4 Fig — Joint states with darker color indicate larger probability. We assumed equal agent parameters: discount factors β1 = β2 = 0.9, reference point b1 = b2 = 0, utility functions u1(x) = u2(x) = x, and weighting function w1(x) = w2(x) = x). (TIF) [file pcbi.1009167.s005.tif]

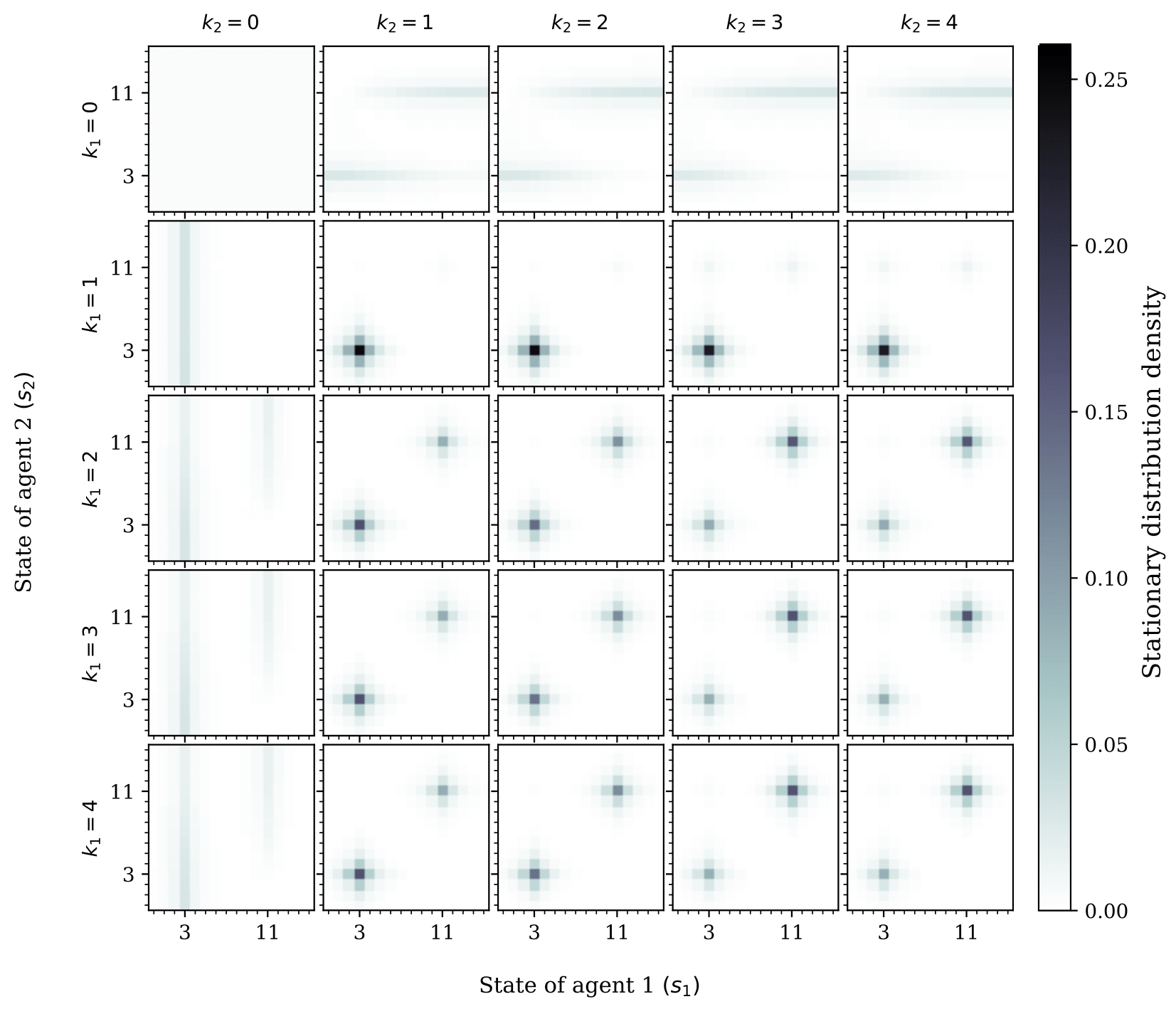

Supplement: S5 Fig — Joint states with darker color indicate larger probability. Here, agent parameters are fixed at: discount factors β1 = β2 = 0.9, reference point b1 = b2 = 0, utility functions u1(x) = u2(x) = x, and weighting functions w1(x) = x, and w2(x)=e-0.5(-log(x))0.9. (TIF) [file pcbi.1009167.s006.tif]

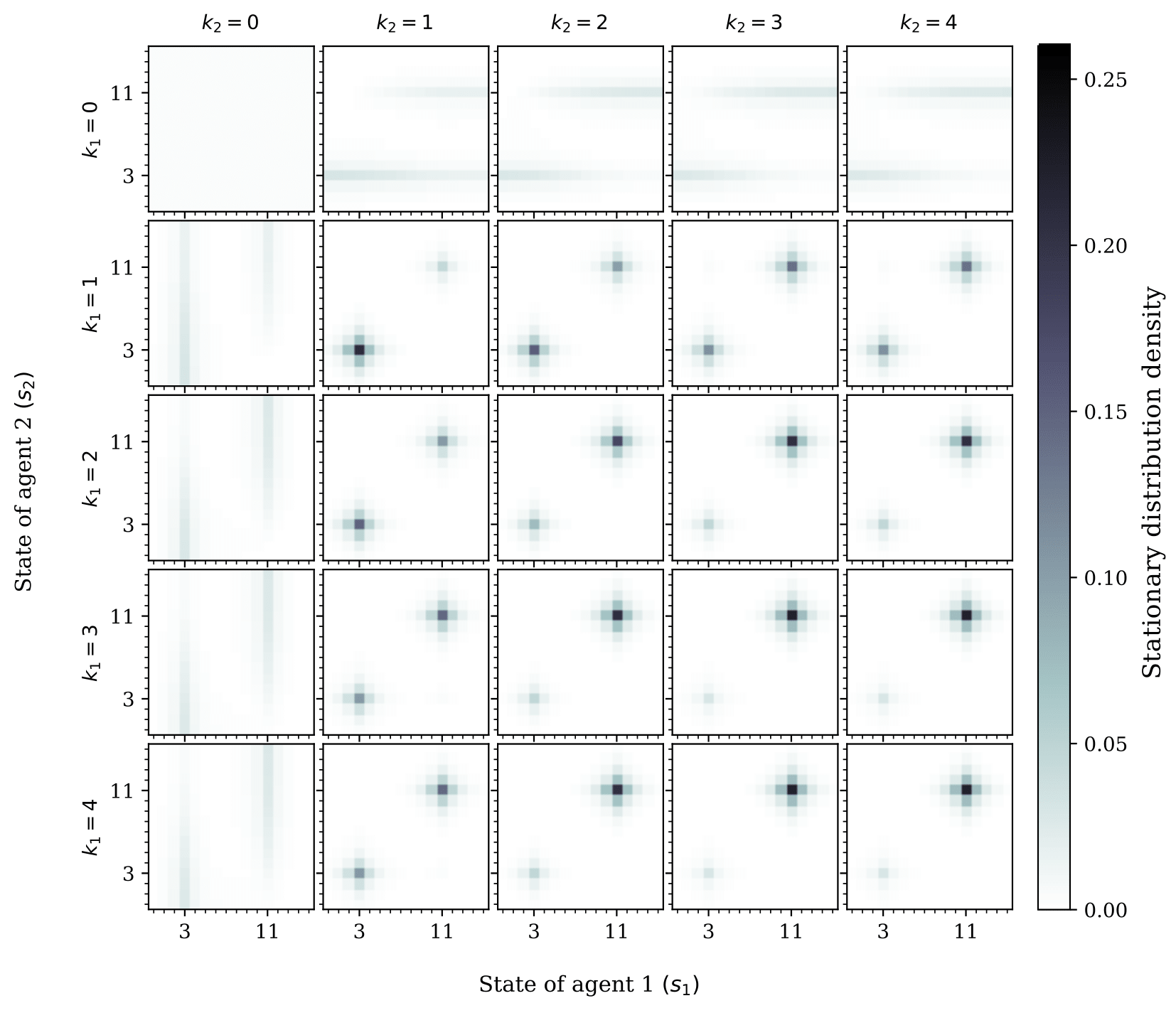

Supplement: S6 Fig — Joint states with darker color indicate larger probability. We assumed equal agent parameters (i.e., discount factors β1 = β2 = 0.9, reference point b1 = b2 = 0, utility functions u1(x) = u2(x) = x0.99, and weighting function w1(x)=w2(x)=e-0.5(-log(x))0.9). (TIF) [file pcbi.1009167.s007.tif]

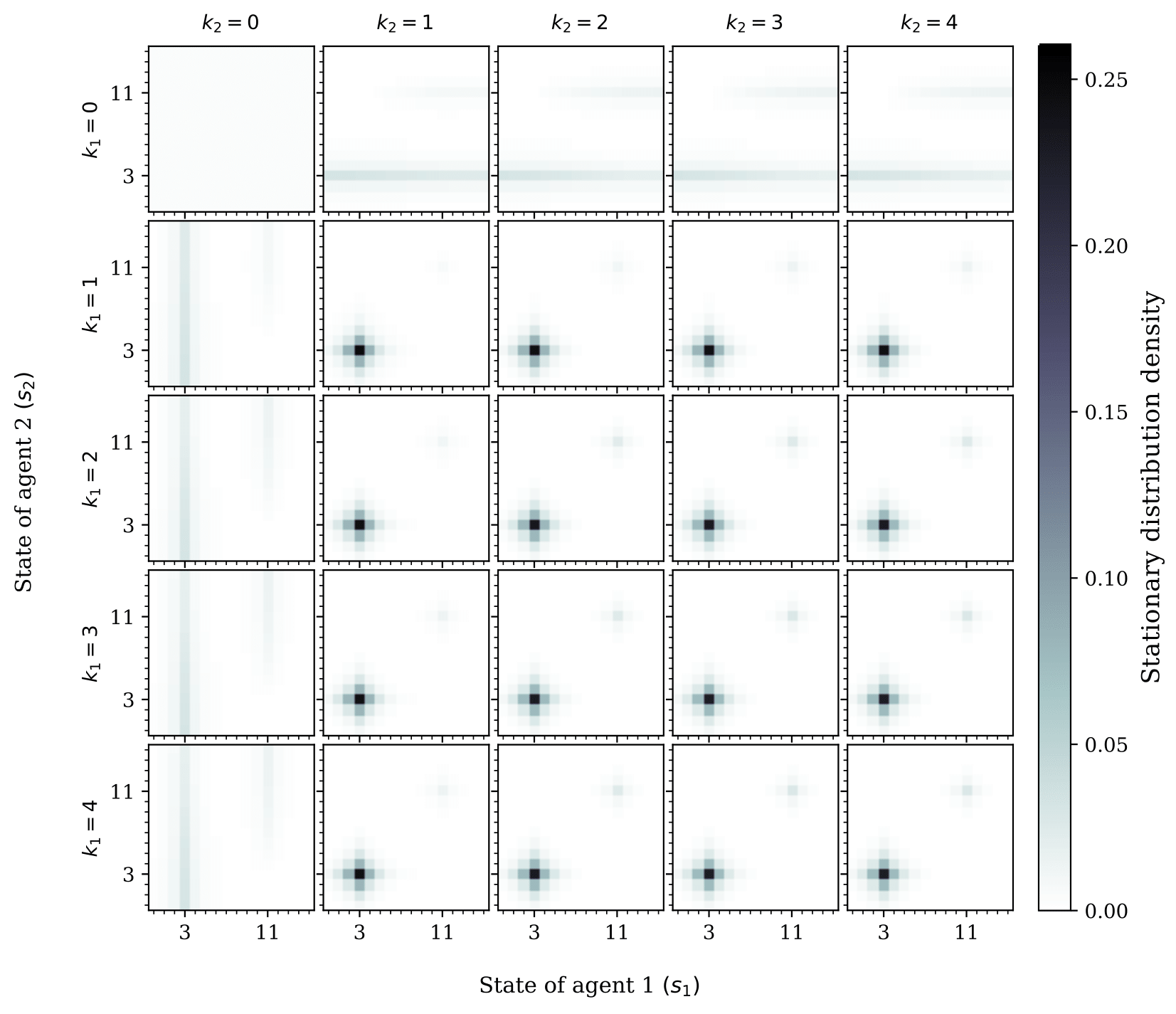

Supplement: S7 Fig — Joint states with darker color indicate larger probability. We assumed equal agent parameters (i.e., discount factors β1 = β2 = 0.9, reference point b1 = b2 = 0, utility functions u1(x) = u2(x) = x0.95, and weighting function w1(x)=w2(x)=e-0.5(-log(x))0.9). (TIF) [file pcbi.1009167.s008.tif]
